# Climate-amplified wildland–urban interface disasters create persistent internal metal mixtures associated with pulmonary impairment

**DOI:** 10.1073/pnas.2613208123

**Published:** 2026-09-28

**Authors:** Alika K. Maunakea, Binh Le, Rafael E. de la Hoz, Alison G. Lee, Zitong Zhang, Martina Kamaka, Marsha Lowery, Lesley Umeda, Brennan Yamamoto, Kathrin Schilling, Ruben Juarez

**Affiliations:** ^a^Department of Anatomy, Biochemistry and Physiology, John A. Burns School of Medicine, Honolulu, HI 96813; ^b^ University of Hawaii Economic Research Organization, University of Hawaii at Manoa, Honolulu, HI 96822; ^c^ Department of Economics, University of Hawaii at Manoa, Honolulu, HI 96822; ^d^ Department of Environmental Medicine, Icahn School of Medicine at Mount Sinai, New York, NY 10029; ^e^ Department of Medicine, Icahn School of Medicine at Mount Sinai, New York, NY 10029; ^f^Department of Native Hawaiian Health, John A. Burns School of Medicine, Honolulu, HI 96813; ^g^ Maui Wildfire Exposure Study, University of Hawaii, Honolulu, HI 96822; ^h^ Department of Environmental Health Sciences, Columbia University, New York, NY 10032

**Keywords:** wildland–urban interface, metal mixtures, environmental biomonitoring, pulmonary function, climate-amplified disasters

## Abstract

Wildland–urban interface (WUI) wildfires are increasingly important climate-related exposure events because they burn built infrastructure and can mobilize legacy contaminants in addition to generating smoke. Although the acute respiratory effects of wildfire smoke are well described, persistent internal metal mixtures and their association with pulmonary function after WUI disasters remain poorly characterized. Using the August 2023 Maui wildfires as a sentinel model, we evaluated 1,400 adults enrolled 6 to 18 mo after the disaster in a community-engaged cohort. We measured 24 urinary metals and assessed pulmonary function by standardized spirometry at the same visit, enabling paired exposure-response analyses. Compared with US reference levels, participants exhibited elevated internal metal burdens, including antimony (40-fold), manganese (3.8-fold), barium (2.3-fold), and arsenic (2.2-fold), with the highest concentrations clustering in the most affected regions. Across three complementary mixture-modeling approaches, higher cumulative metal burden was consistently associated with greater odds of abnormal spirometry. In weighted quantile sum models, each 1-quantile increase in the positive mixture index, driven primarily by arsenic, cadmium, and copper, was associated with higher odds of FVC, FEV_1_, FEV_1_/FVC, and FEF_25–75_ below the lower limit of normal (odds ratio [OR], 1.71, 1.53, 1.83, and 1.84, respectively). These findings suggest that WUI disasters may generate persistent internal metal mixtures associated with pulmonary impairment months after the event and that air monitoring alone may underestimate the longer-term internal toxic burden following climate-amplified WUI disasters.

## Significance

Wildland–urban interface (WUI) disasters create a different exposure problem than wildfires dominated primarily by biomass burning. These disasters also burn built infrastructure and mobilize legacy contaminants, generating exposure profiles that extend beyond acute smoke inhalation. Using the 2023 Maui wildfires as a sentinel case, we found elevated internal metal mixtures 6 to 18 mo after the disaster and observed that higher mixture burden was associated with abnormal pulmonary function. These findings suggest that short-term ambient air monitoring alone may miss an important component of postdisaster toxic burden and support integrating biomonitoring and longer-term respiratory surveillance into WUI disaster response and recovery.

Wildland–urban interface (WUI) wildfires are increasingly frequent climate-amplified disasters that disrupt environmental exposure systems at the population scale (1, 2). Unlike traditional wildland fires, WUI disasters incinerate modern built infrastructure, including treated lumber, electronics, and vehicles, while also mobilizing historically deposited contaminants from former agricultural and industrial land use. As a result, these events generate multisource exposure environments that extend beyond acute smoke inhalation and may persist long after flames are extinguished (3–9). Metals such as arsenic, cadmium, lead, and antimony have established direct and indirect toxicological links to oxidative stress, systemic inflammation, cardiopulmonary disease, and impaired lung function (10–13). Yet population-level evidence connecting postdisaster internal metal burden to measurable respiratory impairment remains limited, particularly in structurally vulnerable communities, including Indigenous and geographically isolated populations.

WUI wildfires should therefore be understood not only as acute smoke events, but as coupled contamination events that can generate persistent internalized mixtures from both structural combustion and mobilized legacy pollutants. This distinction matters because vegetation-only fire models may not capture the broader toxicological profile of disasters that burn homes, vehicles, electrical systems, and other components of the built environment (3–6). It also suggests that ambient air monitoring, although essential, may provide an incomplete picture of postdisaster health risk if internal dose persists after visible smoke has cleared or if residents continue to be re-exposed through ash-contaminated soil, settled dust, debris, and other environmental media during recovery (8, 9). As these disasters increase in frequency and severity across climate-vulnerable regions, understanding their longer-term biological footprint has important implications for disaster surveillance, exposure science, and recovery planning.

In August 2023, catastrophic wildfires on the island of Maui, Hawaiʻi, destroyed entire communities and displaced residents across the island (14, 15). The Maui Wildfire Exposure Study (MauiWES) was established as a community-engaged cohort to evaluate the long-term biological and environmental consequences of this disaster through biomonitoring, clinical assessment, and longitudinal follow-up. Early environmental assessments documented elevated concentrations of multiple metals in ash and surface samples across burn and downwind zones (15). The Maui fires were fast-moving WUI disasters involving both wildland fuels and extensive built-environment combustion. They also occurred in a setting where postfire cleanup, ash redistribution, and re-entrainment of contaminated dust plausibly extended exposure beyond the acute fire period. We therefore treat Maui as a sentinel case for a broader class of destructive WUI fires in which structural combustion and legacy contamination may jointly shape longer-term human exposure. However, whether these disaster-perturbed exposure conditions translated into sustained internal metal burden and measurable respiratory health impacts among residents remained unclear.

Even though environmental metals rarely occur in isolation, only a few epidemiologic studies have evaluated metal mixtures and respiratory outcomes (12, 14, 15). In post-WUI settings, exposures are inherently multicomponent, reflecting overlapping contributions from combustion residues, redistributed soil contaminants, and other environmental sources (16, 17). Because correlated metals may act through shared toxicological pathways, single-exposure models may underestimate cumulative risk. Mixture-based approaches provide a framework for evaluating this joint burden and for identifying the components that most strongly shape the overall exposure profile (18–20).

We therefore quantified urinary metal concentrations as indicators of internalized, subchronic exposure and examined their associations with spirometry-defined pulmonary function among adults enrolled in the MauiWES baseline cohort. The respiratory system was prioritized because of its vulnerability to inhaled particulate and metal-rich debris and the well-established cardiopulmonary effects of wildfire smoke exposure (16, 21, 22). Using Maui as a sentinel model, we tested whether adults living in a post-WUI disaster environment exhibit persistent internal metal mixtures and whether higher mixture burden is associated with abnormal pulmonary function.

## Results

### Cohort Characteristics.

**Fig. 1.**
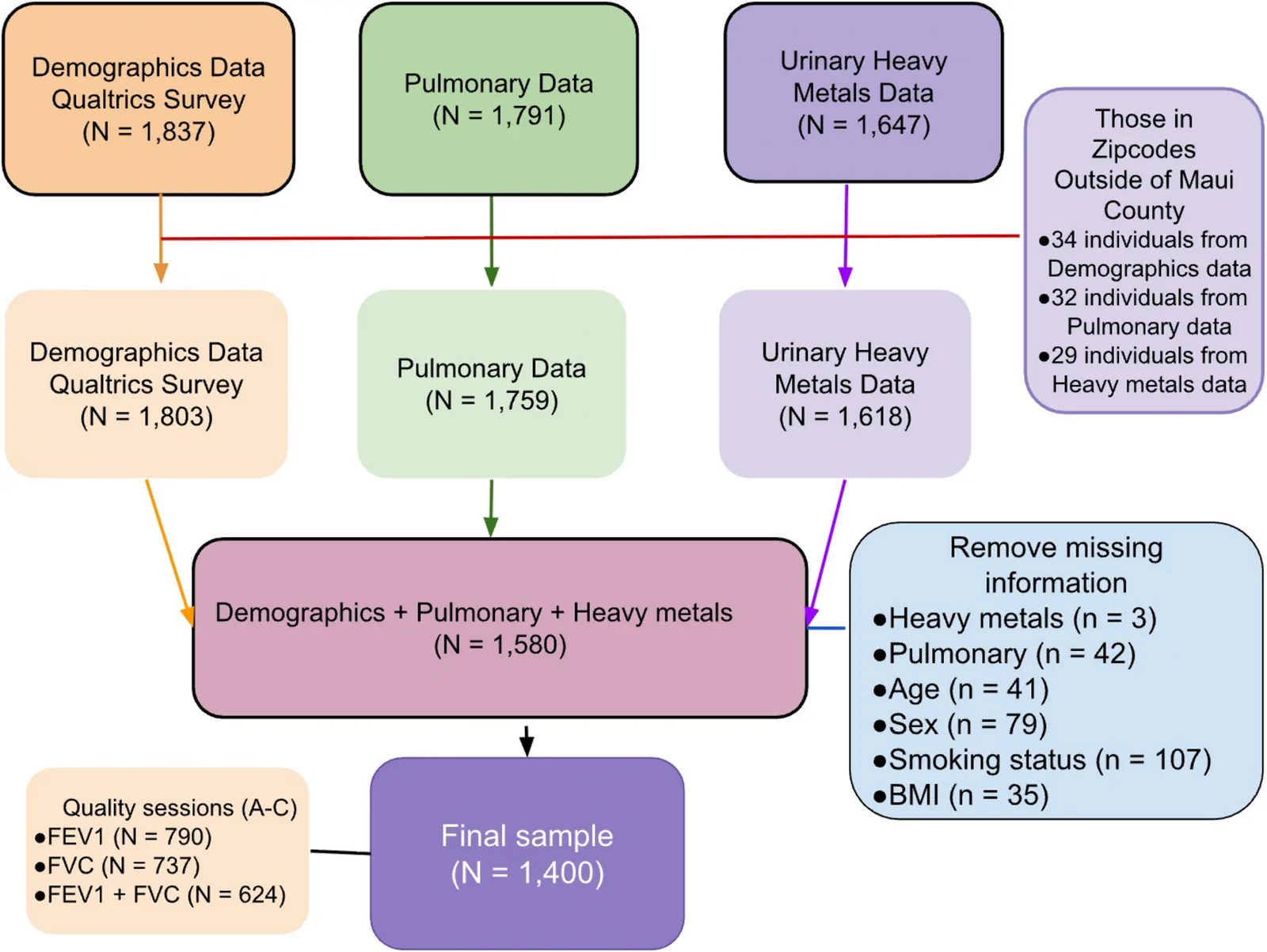
Flow diagram of sample construction for the MauiWES analytic dataset. Illustration of the integration of demographic survey data, pulmonary function testing (spirometry), and urinary metal measurements; the exclusion of participants residing in ZIP codes outside Maui County; and stepwise removal of records missing key covariates (metals, pulmonary outcomes, age, sex, smoking status, and BMI). After all exclusions, the final analytic sample included 1,400 participants. After restriction to ATS/ERS spirometry quality grades A–C, pulmonary outcome-specific sample sizes were N = 790 for FEV_1_ < LLN, N = 737 for FVC < LLN, and N = 624 for FEV_1_/FVC < LLN and FEF_25–75_ < LLN.

A total of 1,837 adults residing across Maui County enrolled in the MauiWES baseline between January 2024 and February 2025 (*SI Appendix*, Fig. S1), corresponding to approximately 6 to 18 mo after the 2023 Maui wildfires (23, 24). Spirometry data underwent centralized quality review by trained technicians and independent external reviewers. Only tests meeting ATS/ERS quality grades A–C were included in analytic models, as specified. Of the enrolled participants, 1,791 completed spirometry and 1,647 provided urine specimens for metal analysis. Among spirometry tests meeting quality criteria, 790 participants contributed valid FEV_1_ measurements, 737 contributed valid FVC measurements, and 624 contributed valid FEV_1_/FVC measurements. After excluding participants residing outside Maui County and those missing urinary metals or key covariates, the analytic cohort comprised 1,400 individuals (Fig. 1), from whom 27 urinary metals were measured, with 24 retained for analysis (*SI Appendix*, Table S1). Outcome-specific sample sizes varied according to spirometry availability and are noted where relevant.

Participants had a median age of 47 y (IQR 36 to 60), and 60.9% were female (*SI Appendix*, Table S2). The cohort reflected the demographic composition of affected communities, including Native Hawaiian or other Pacific Islander (20.2%), Filipino (18.9%), Hispanic/Latino (17.5%), non-Filipino Asian (8.6%), and White (29.1%) residents. Current tobacco use (i.e., smoking) was reported by 16.8%, and the median body mass index (BMI) was 27.9 kg/m^2^ (IQR 24.1 to 32.7). Self-reported exposure to wildfire smoke or ash varied substantially by region. In Lahaina, more than 40% of participants reported daily (21.3%) or weekly (19.9%) exposure to wildfire ash, closely paralleled by Kula (21.8% daily; 17.9% weekly). In contrast, Kihei and Wailuku/Kahului had much lower high-frequency exposure, with daily exposure reported by only 9.3% and 8.8%, respectively, and Wailuku/Kahului showing the highest proportion reporting no exposure (34.4%). These regional differences were highly significant (χ^2^ = 130.38, *P* = 9.9 × 10^–24^). In contrast, the time elapsed from the fires to data collection did not differ meaningfully across regions. Median months since the August 2023 fires were similar, with residents of Kihei 12.3 mo (IQR 11.9 to 13.0), Kula 11.9 (6.3 to 13.0), Lahaina 10.7 (6.3 to 16.0), and Wailuku/Kahului 13.0 (12.4 to 16.0) showing no statistically significant regional variation (Kruskal–Wallis *P* > 0.10).

### Geographic Distribution of Pulmonary Impairment.

**Fig. 2.**
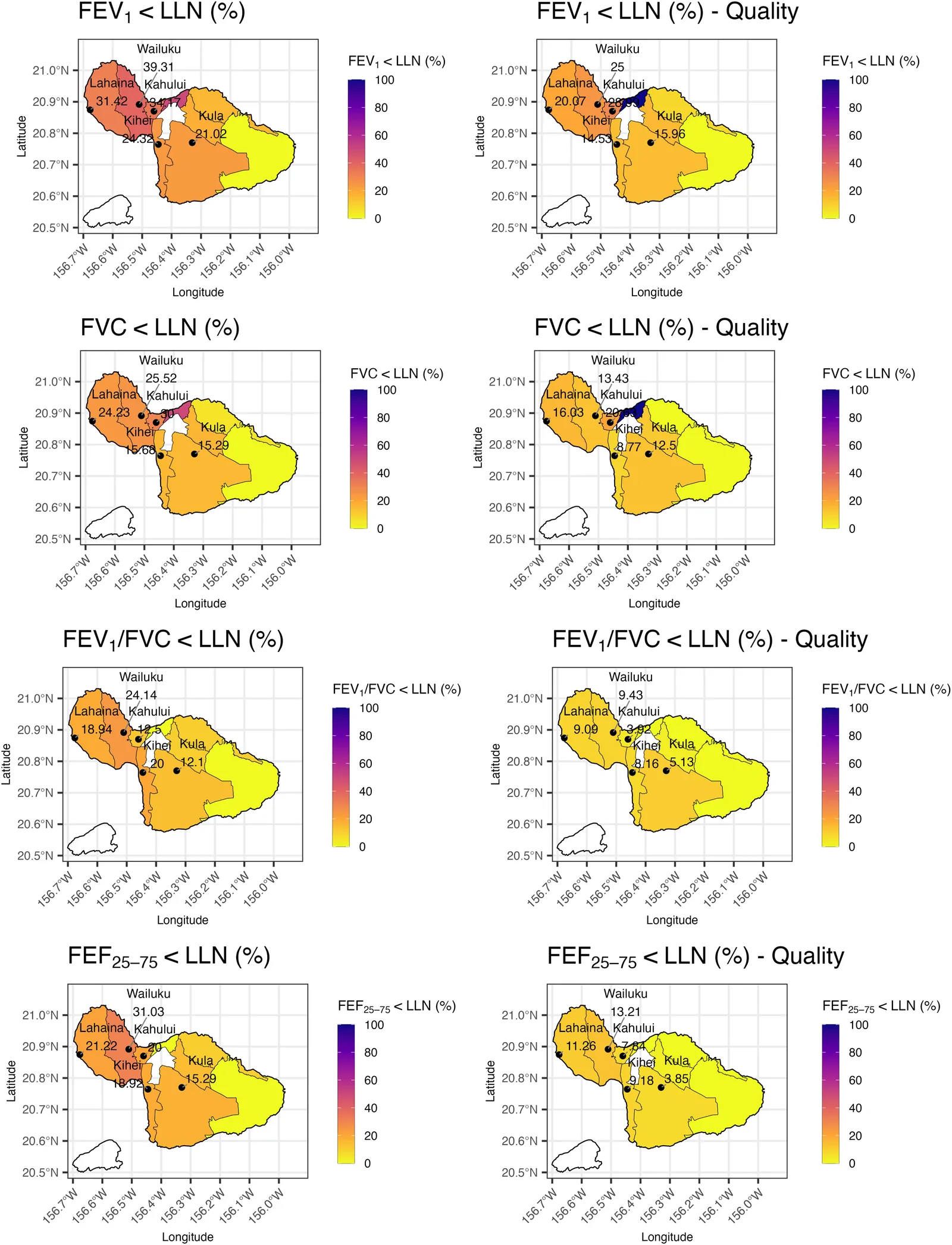
Geographic distribution of abnormal pulmonary function among MauiWES participants. Each row displays the percentage of individuals with values below the LLN for a given spirometry measure of pulmonary function: FEV_1_, FVC, FEV_1_/FVC, and FEF_25–75_. The *Left* panel in each row presents LLN prevalence based on all available spirometry tests (N = 1,759). The *Right* panel presents LLN prevalence restricted to tests meeting ATS/ERS quality grades A–C. Percentages were mapped across major population regions of Maui, including Lahaina, Kihei, Kahului, Wailuku, and Kula. Sample sizes of session quality varied by outcome: FEV_1_ < LLN (N = 968), FVC < LLN (N = 881), FEV_1_/FVC < LLN (N = 742), and FEF_25–75_ < LLN (N = 742). Color scales represent the proportion of abnormal results, with higher values indicating greater prevalence of pulmonary impairment.

Descriptive prevalence estimates are presented for all interpretable spirometry sessions, whereas inferential analyses were restricted to tests meeting American Thoracic Society/European Respiratory Society (ATS/ERS) quality grades A–C. Among participants with interpretable spirometry, 23.0% exhibited FVC values below the lower limit of normal (LLN), 30.6% were below LLN for FEV_1_, 18.4% for the FEV_1_/FVC ratio, and 21.2% for FEF_25–75_. These abnormalities were not evenly distributed geographically (*SI Appendix*, Table S3). Kula, located at higher elevation and characterized by greater topographic openness, consistently demonstrated the lowest burden of pulmonary impairment, with FVC < LLN in 15.3% and FEV_1_ < LLN in 21.0% of residents. In contrast, Wailuku/Kahului exhibited the highest prevalence across nearly all spirometry metrics, including 27.6% with reduced FVC and 37.0% with reduced FEV_1_, corresponding to approximately 1.7-fold and 1.8-fold higher frequencies relative to Kula, respectively. Lahaina, a coastal region and historic town directly affected by the wildfire and characterized by the highest reported levels of smoke and ash exposure, also exhibited a high prevalence of pulmonary impairment (24.2% for FVC < LLN and 31.4% for FEV_1_ < LLN). In contrast, Kihei, another coastal community located approximately 15 miles south of Lahaina, showed intermediate prevalence levels across spirometry outcomes. The geographic pattern of impaired lung function paralleled the regional gradient in self-reported smoke and ash exposure, with the highest burden concentrated in Lahaina and Wailuku/Kahului and the lowest in Kula. Similar regional patterns were observed when analyses were restricted to ATS/ERS quality grade A–C sessions, indicating that the geographic gradients were not attributable to differential test quality. Spatial mapping of spirometric impairment prevalence further illustrated this correspondence, revealing clusters of reduced lung function in Lahaina and Wailuku/Kahului and minimal impairment prevalence in Kula (Fig. 2).

### Geographic Distribution of Urinary Metal Exposures.

**Fig. 3.**
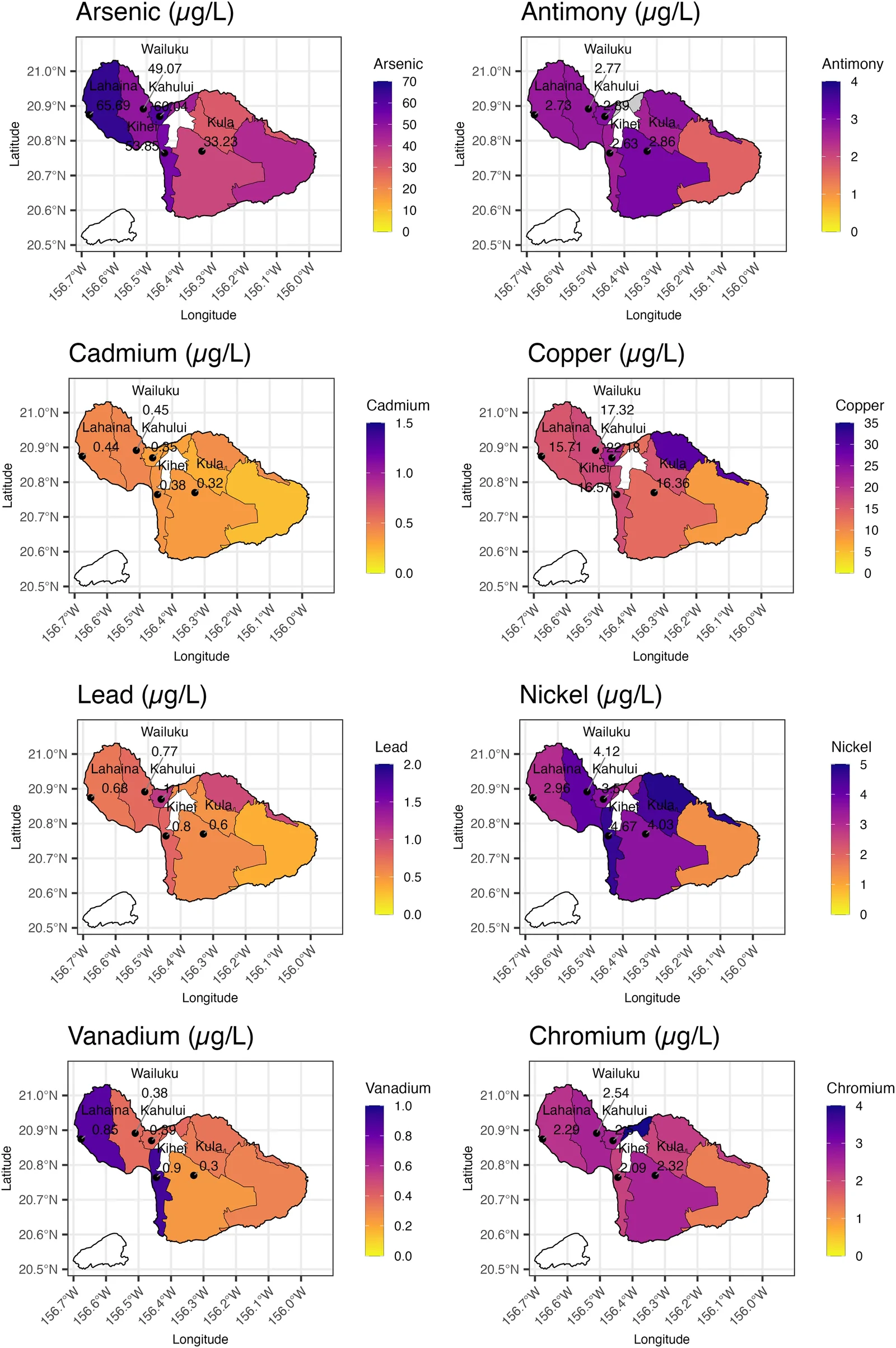
Geographic distribution of urinary metal concentrations among MauiWES participants. Maps display mean urinary concentrations (µg/L), normalized for urine specific gravity, for 8 (of 24) metals measured in MauiWES: arsenic, antimony, cadmium, copper, lead, nickel, vanadium, and chromium. Blank areas indicate missing regional information. Based on participants with available urinary metal data (analytic N = 1,618), regional values are shown for five primary regions on Maui: Lahaina (N = 1,041), Kula (N = 139), Kahului (N = 117), Kihei (N = 182), and Wailuku (N = 139). Color gradients represent region-level mean concentrations, with darker intensities indicating higher levels, illustrating the geographic heterogeneity in exposure profiles (see *SI Appendix*, Fig. S2 for all metals measured).

Among the 1,618 participants with urinary biomonitoring data, metal concentrations differed significantly across regions. Spatial interpolation maps demonstrated clear geographic gradients across the island (Fig. 3 and *SI Appendix*, Fig. S2). Lahaina showed high-intensity concentrations for arsenic, thallium, cadmium, and vanadium. Kula displayed elevated calcium, magnesium, chromium, and lithium. Wailuku/Kahului exhibited higher levels of copper, iron, and molybdenum, while Kihei generally showed lower-intensity values across the metal panel. Quantitative comparisons across regions corroborated these spatial patterns (*SI Appendix*, Table S4). Arsenic was high in Lahaina (median 22.34 µg/L; IQR 9.83 to 55.40), exceeding concentrations in Kihei (19.69 µg/L; IQR 8.16 to 46.52) and Kula (17.82 µg/L; IQR 7.76 to 36.96), and similar to Wailuku/Kahului (22.54 µg/L; IQR 10.58 to 65.05) (*P* = 0.010). Lahaina also showed high medians for cadmium (0.29 µg/L), cobalt (0.39 µg/L), nickel (2.37 µg/L), and vanadium (0.24 µg/L), with levels also varying by region. Several metals reached their highest concentrations outside Lahaina. Copper was greatest in Wailuku/Kahului (12.53 µg/L; IQR 8.77 to 19.89) and Kihei (11.91 µg/L; IQR 8.46 to 17.13), exceeding levels observed in Lahaina and Kula (both approximately 10.5 µg/L) (*P* < 0.001). Iron (18.46 µg/L) and molybdenum (59.43 µg/L) also peaked in Wailuku/Kahului (*P* = 0.031 and *P* = 0.003). Nickel, cesium, and barium exhibited significant regional heterogeneity, with Kihei showing the highest nickel (3.89 µg/L; *P* < 0.001), while barium was greatest in Kula (3.52 µg/L; *P* = 0.010). Thallium differed significantly across regions (*P* < 0.001), with Lahaina and Kula having higher medians (both 0.20 µg/L) than Kihei (0.19 µg/L) or Wailuku/Kahului (0.17 µg/L). Lead medians were more uniform (0.46 to 0.49 µg/L) but approached regional, less conservative significance (*P* = 0.095).

Multivariate analyses identified clear regional clustering of metal profiles. Radar plots and heatmaps of standardized concentrations showed higher relative intensities of arsenic, cadmium, nickel, cobalt, vanadium, and thallium in Lahaina; elevated calcium, magnesium, chromium, and lithium in Kula; and higher copper, iron, and molybdenum in Wailuku/Kahului (*SI Appendix*, Fig. S3). Kihei displayed generally lower or mid-range standardized values across most metals. Principal component analyses (PCA) further delineated these regional patterns (*SI Appendix*, Fig. S4). In Lahaina, the first principal component (PC1) exhibited high loadings for arsenic, selenium, cadmium, copper, zinc, and thallium and explained 15.5% of the variance in that region. In Kula, PC1 loaded strongly on calcium, magnesium, chromium, and molybdenum, explaining 15.0% of the variance. In Wailuku/Kahului, copper, zinc, iron, and antimony contributed substantially to PC1, explaining 13.7% of the variance. Kihei showed smaller and more broadly distributed loadings with no dominant metal drivers.

### Comparison of Urinary Metal Concentrations with National Reference Values.

To contextualize postwildfire exposures, we compared urinary metals from MauiWES with US reference levels from NHANES 2011–2012 using uncorrected raw urine metal concentrations for both datasets (Table 1). The mean and percentile distribution of metal concentrations are presented in *SI Appendix*, Table S5. Median concentrations for most metals were significantly higher in MauiWES, indicating that wildfire-related contamination contributed to sustained internal exposure months after the disaster. The most pronounced elevation occurred for antimony, which was nearly 40-fold higher in MauiWES than in NHANES (1.97 vs. 0.05 µg/L; *P* < 0.001). Manganese was also markedly elevated (3.8-fold, 0.46 vs. 0.12 µg/L; *P* < 0.001). Several additional metals exceeded NHANES background levels by ≥1.5-fold and demonstrated strong statistical significance, including barium (2.3-fold, *P* < 0.001), arsenic (2.2-fold, *P* < 0.001), and tungsten (2.6-fold, *P* < 0.001). These metals represent the most substantial deviations from national reference values and closely correspond to those enriched in wildfire ash and debris. Moderate but statistically robust elevations were also observed for cesium (1.4-fold, *P* < 0.001), strontium (1.4-fold, *P* < 0.001), lead (1.8-fold, *P* < 0.001), and molybdenum (1.1-fold, *P* < 0.001). Although these increases were smaller in magnitude, the consistent statistical significance and the toxicological relevance of several of these metals, particularly lead, cesium, and strontium, warrant their inclusion. By contrast, tin (1.05-fold, *P* = 0.009), thallium (1.13-fold, *P* = 0.004), and cobalt (1.13-fold, *P* = 0.041) showed small but statistically significant differences that fall below thresholds typically considered indicative of meaningful population-level exposure shifts. These metals are therefore reported for completeness but not interpreted as primary drivers of exposure differences. Cadmium showed no significant difference between MauiWES and NHANES (*P* = 0.40). The MauiWES cohort also exhibited substantially lower limits of detection (LoDs) than the NHANES cohort (*SI Appendix*, Table S6). Across metals, the overall pattern demonstrates that Maui residents, particularly those in Lahaina, experienced internal metal burdens that were substantially elevated relative to the general US population, with several metals exceeding NHANES distributions by 2- to 40-fold.

**Table 1.** Comparison of urinary concentrations of matched metals between MauiWES (2024–2025) and NHANES (2011–2012)

Metal concentrations (µg/L)	NHANES 2011–2012	MauiWES 2024–2025	*P*-value
Barium	1.11 (0.55-2.18)	2.56 (1.67-3.93)	<0.001
Missing/Not reported	60	0	
Cadmium	0.22 (0.11-0.44)	0.23 (0.12-0.47)	0.4
Missing/Not reported	60	0	
Cobalt	0.31 (0.19-0.49)	0.35 (0.23-0.55)	0.041
Missing/Not reported	60	0	
Cesium	4.21 (2.55-6.32)	5.87 (3.48-8.42)	<0.001
Missing/Not reported	61	0	
Molybdenum	41.10 (21.60-69.20)	46.05 (23.35-85.07)	<0.001
Missing/Not reported	60	0	
Manganese	0.12 (0.06-0.20)	0.46 (0.35-0.66)	<0.001
Missing/Not reported	60	0	
Lead	0.22 (0.06-0.61)	0.39 (0.24-0.67)	<0.001
Missing/Not reported	60	0	
Antimony	0.05 (0.03-0.08)	1.97 (1.77-2.22)	<0.001
Missing/Not reported	60	0	
Tin	0.56 (0.29-1.22)	0.59 (0.27-1.35)	0.009
Missing/Not reported	60	0	
Strontium	89.50 (49.38-161.60)	125.35 (63.99-199.10)	<0.001
Missing/Not reported	61	0	
Thallium	0.16 (0.10-0.25)	0.18 (0.10-0.27)	0.004
Missing/Not reported	60	0	
Tungsten	0.07 (0.03-0.14)	0.18 (0.12-0.28)	<0.001
Missing/Not reported	70	0	
Arsenic	8.09 (3.89-18.62)	18.10 (7.73-48.39)	<0.001
Missing/Not reported	90	3	

Values are presented as median (IQR). Two-sided *t* tests were used to compare distributions between cohorts. NHANES includes 1,852 adults with urinary metal measurements, and MauiWES includes 1,647 adults. Analyses were restricted to participants aged ≥18 y and excluded pregnant individuals. The table includes only metals available in both datasets. Concentrations reflected raw urinary values and were not corrected for specific gravity or creatinine to ensure comparability across cohorts.

### Single Metal Associations and BKMR Exposure–Response Functions.

We next evaluated whether individual urinary metals were associated with abnormal spirometry. In generalized linear models adjusted for age, sex, smoking status, BMI, and residential region, several metals demonstrated significant associations with impaired pulmonary function (*SI Appendix*, Fig. S5 and Table S7). Although effect sizes varied across outcomes, the strongest positive associations were observed for metals previously implicated in combustion-derived toxicity. Arsenic was significantly associated with FVC < LLN (OR = 1.26; 95% CI: 1.06 to 1.51), while cadmium showed one of the highest single metal effects, corresponding to nearly a twofold increase in the odds of FEF_25–75_ < LLN (OR = 1.76; 95% CI: 1.11 to 2.77) and a borderline association with reduced FEV_1_ (OR = 1.34; 95% CI: 1.01 to 1.76). Higher concentrations of lead were associated with more than a twofold increase in the odds of FEV_1_/FVC < LLN (OR = 2.20; 95% CI: 1.33 to 3.65). Other metals demonstrated heterogeneous associations, including inverse relationships for lithium, calcium, thallium, and cobalt, reflecting the diverse and complex biological functions represented within the metal panel. We next applied BKMR to examine continuous exposure–response relationships and potential nonlinearities (*SI Appendix*, Fig. S6). Univariate exposure–response functions revealed monotonic or near monotonic increases in predicted impairment with rising concentrations of several metals. Arsenic showed steadily increasing predicted probabilities of both impaired FVC and FEV_1_ deficits, while cadmium exhibited similar monotonic increases across small- and large-airway outcomes, particularly FEF_25–75_. Copper showed positive, approximately linear exposure–response curves across multiple outcomes, consistent with its elevated weights in mixture models. Lead demonstrated a positive, nonlinear association for the FEV_1_/FVC ratio, with the steepest increase in predicted risk of obstructive impairment at higher exposure levels. BKMR univariate exposure–response functions did not suggest strong nonlinearity or threshold effects within the observed exposure distributions.

### Overall Effects of Metal Mixtures on Pulmonary Function.

**Fig. 4.**
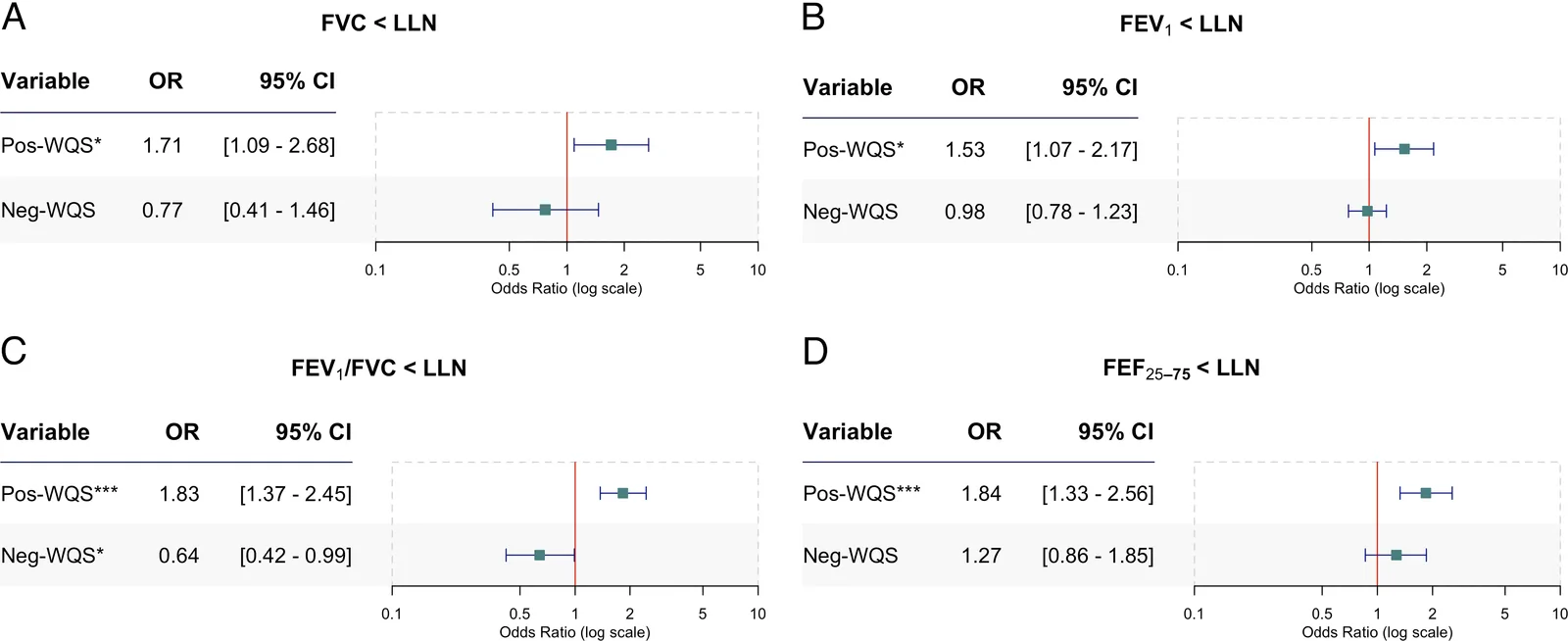
Associations between metal mixture exposure and abnormal pulmonary function estimated using dual-index WQS regression. Panels depict associations with (*A*) FVC < LLN, (*B*) FEV_1_ < LLN, (*C*) FEV_1_/FVC < LLN, and (*D*) FEF_25–75_ < LLN. Odds ratios (ORs) and 95% CIs represent the association between metal mixture indices and the likelihood of spirometry measures falling below the LLN. Models were adjusted for age, sex, smoking status, BMI, and restricted to spirometry tests meeting ATS/ERS quality grades A–C. Sample sizes differed by outcome: FVC < LLN (N = 737), FEV_1_ < LLN (N = 790), and FEV_1_/FVC < LLN and FEF_25–75_ < LLN (N = 624). Statistical significance is indicated as follows: +*P* < 0.10; *P < 0.05; **P < 0.01; ***P < 0.001.

**Fig. 5.**
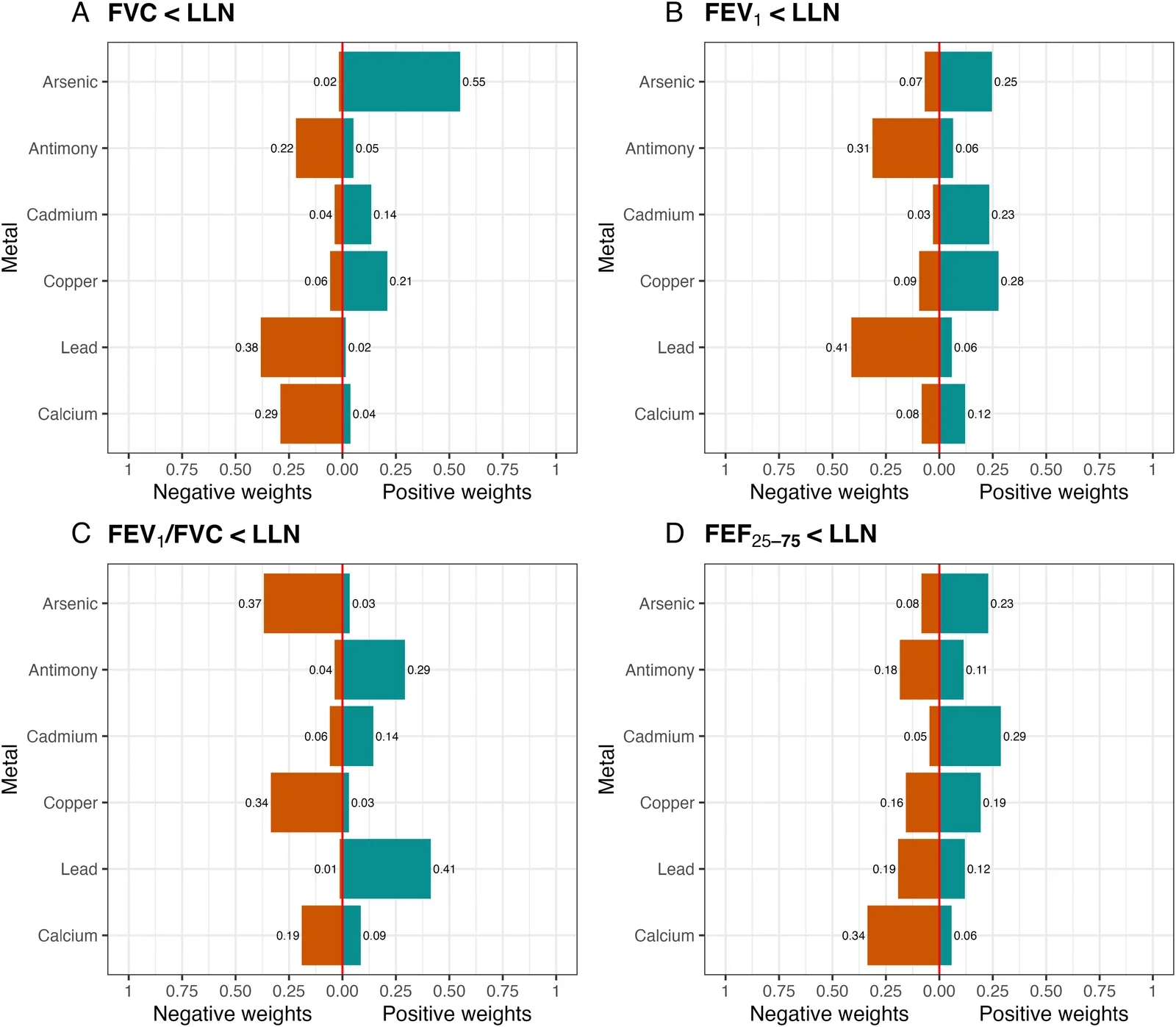
Metal-specific weight contributions to pulmonary outcomes in dual-index WQS regression models. Panels depict the relative contribution and directionality of individual metals associated with (*A*) FVC < LLN, (*B*) FEV_1_ < LLN, (*C*) FEV_1_/FVC < LLN, and (*D*) FEF_25–75_ < LLN. Bars indicate estimated weights assigned to each metal within positive (*Right*) and negative (*Left*) mixture indices, with direction defined by the dominant absolute weight contribution. Models were adjusted for age, sex, smoking status, BMI, and restricted to spirometry tests meeting ATS/ERS quality grades A–C. Sample sizes differed by outcome: FVC < LLN (N = 737), FEV_1_ < LLN (N = 790), and FEV_1_/FVC < LLN and FEF_25–75_ < LLN (N = 624).

Because wildfire-related metals co-occur and often act through shared toxicological pathways (11), we next evaluated the joint contribution of multiple metals using two complementary mixture modeling approaches. In dual-index WQS models, each 1-quantile increase in the positive mixture index was associated with higher odds of abnormal pulmonary function across all four pulmonary outcomes (Fig. 4). Associations were highest for FEF_25–75_ < LLN (OR = 1.84; 95% CI: 1.33 to 2.56), FEV_1_/FVC < LLN (OR = 1.83; 95% CI: 1.37 to 2.45), and FEV_1_ < LLN (OR = 1.53; 95% CI: 1.07 to 2.17). The association with FVC < LLN was similarly positive (OR = 1.71; 95% CI: 1.09 to 2.68). These effect sizes, which exceed those observed for most individual metals, indicate that the cumulative metal mixture appears to have an even more pronounced association with pulmonary impairment risk than any single exposure component. Mixture weights identified arsenic, cadmium, copper, and antimony as the dominant contributors to the positive direction of the WQS index, together accounting for more than half of the total positive weight in most outcomes (Fig. 5). In contrast, lead and calcium contributed the highest negative weights, reflecting complex intermetal correlations and the ability of WQS to accommodate both risk-enhancing and risk-attenuating components within the mixture. Because mixture weights reflect conditional contributions within a correlated exposure set, negative weights should not be interpreted as evidence that these metals are protective in isolation. Q-gComp with or without bootstrapping yielded highly concordant results. Each 1-decile increase in the joint exposure index was associated with greater odds of FEV_1_ < LLN (OR = 1.79; 95% CI: 1.22 to 2.62) and FEF_25–75_ < LLN (OR = 2.46; 95% CI: 1.30 to 4.66) with similar, albeit weaker, positive associations observed for other outcomes (*SI Appendix*, Fig. S7). Q-gComp weight distributions reinforced the importance of arsenic and copper as key positive contributors, while again identifying lead as the highest negative-weight component for FVC and FEV_1_ outcomes (*SI Appendix*, Fig. S8). Further, BKMR posterior inclusion probabilities identified arsenic, cadmium, and lead as consistently influential contributors across outcomes (*SI Appendix*, Fig. S9), reaffirming their roles as key components of the multimetal mixture most significantly associated with impaired lung function.

### Region-Specific Mixture Effects on Pulmonary Function.

Given the pronounced spatial heterogeneity in metal exposures, we next evaluated mixture–pulmonary associations by wildfire-impacted and nonimpacted areas. In Lahaina/Kula, standard WQS regression yielded a coherent and stable positive-direction mixture structure, consistent with the Q-gComp results (*SI Appendix*, Tables S8 and S9). Each 1-quantile increase in the WQS index was associated with significantly higher odds of FEV_1_/FVC < LLN (OR = 1.55; 95% CI: 1.09 to 2.18), FEV_1_ < LLN (OR = 1.32; 95% CI: 1.03 to 1.69) and FEF_25–75_ < LLN (OR = 1.72; 95% CI: 1.26 to 2.33). The association with FVC < LLN was also positive but not statistically significant. Q-gComp models showed a parallel pattern, with a significant association for FEF_25–75_ < LLN (OR = 1.89; 95% CI: 1.06 to 3.36) and positive but nonsignificant estimates for FVC, FEV_1_, and FEV_1_/FVC. The principal positive weight contributors in this region were arsenic, antimony, cadmium, copper, nickel, and vanadium, which together accounted for the full positive direction mixture structure across all outcomes (*SI Appendix*, Fig. S10). As shown in *SI Appendix*, Tables S8 and S9, in Wailuku/Kahului/Kihei, Q-gComp produced a more stable and interpretable mixture weight structure than WQS, and these weights were therefore used to characterize mixture composition. The regional mixture was significantly associated with FEF_25–75_ < LLN (OR = 3.33; 95% CI: 1.27 to 8.72), with positive but nonsignificant associations for FVC, FEV_1_, and FEV_1_/FVC. WQS results were directionally consistent and showed significant associations for FVC < LLN (OR = 2.52; 95% CI: 1.39 to 4.55), FEV_1_ < LLN (OR = 1.98; 95% CI: 0.99 to 3.94), and FEF_25–75_ < LLN (OR = 2.70; 95% CI: 1.73 to 4.20). The dominant positive weight contributors included arsenic, cadmium, copper, and iron, while thallium and selenium contributed negative weights (*SI Appendix*, Fig. S11).

### Sensitivity Analyses.

Because active tobacco use is a major determinant of pulmonary impairment, we restricted analyses to nonsmokers. Mixture estimates from both dual-index WQS and Q-gComp in this subsample remained consistent in direction and magnitude with the full-cohort results (*SI Appendix*, Fig. S12). Among nonsmokers, the WQS mixture index showed significant associations with FVC < LLN (OR = 1.55; 95% CI: 1.17 to 2.05), FEV_1_ < LLN (OR = 1.90; 95% CI: 1.11 to 3.24), and FEF_25–75_ < LLN (OR = 1.72; 95% CI: 1.35 to 2.20). Q-gComp likewise identified significant associations for FEV_1_ < LLN (OR = 1.43; 95% CI: 1.03 to 2.00) and FEF_25–75_ < LLN (OR = 1.98; 95% CI: 1.15 to 3.43), confirming that the observed mixture–pulmonary associations were not confounded by current smoking. The mixture weights derived from WQS remained largely unchanged, with arsenic, cadmium, and copper consistently emerging as the dominant positive contributors (*SI Appendix*, Fig. S13). This stability indicates that the primary mixture structure is not dependent on the smoking distribution within the cohort. Because participants were enrolled at varying times after the wildfire, we performed a sensitivity analysis adjusting for months since the fire to account for temporal variation in postfire exposure (*SI Appendix*, Figs. S14–S16). The significant associations between mixture burden and abnormal pulmonary function remained consistent with the primary findings, and the relative weight contributions and directions of the major metals were largely unchanged, indicating that the primary mixture–pulmonary associations were not driven by sample collection timing. In addition, we conducted quality assurance and quality control sensitivity analyses to evaluate the potential contribution of urine collection and aliquoting materials to measured urinary metal concentrations (*SI Appendix*, Tables S10 and S11). These analyses demonstrated that contamination arising from collection tubes, storage vials, and laboratory processing materials was negligible relative to the concentrations observed in participant samples. Collectively, these findings support that the measured metal concentrations primarily reflect biological exposure rather than artifacts introduced during sample collection, handling, storage, or analytical processing.

## Discussion

This study suggests that WUI disasters create a distinct postdisaster exposure regime characterized by persistent internal metal mixtures associated with pulmonary impairment months after the event. In this community-engaged cohort, adults living in a post-WUI disaster environment exhibited elevated internal metal burdens 6 to 18 mo after the August 2023 Maui fires, and a higher cumulative mixture burden was consistently associated with abnormal spirometry. By integrating urinary metal biomonitoring, standardized spirometry, and complementary mixture-modeling approaches, these findings extend prior MauiWES analyses documenting persistent cardiopulmonary and psychosocial impacts by identifying internalized metal mixtures as a plausible contributor to postdisaster respiratory risk (23, 24). More broadly, the results support the view that climate-amplified WUI disasters may produce prolonged toxic exposure environments that are not adequately captured by an acute, smoke-only wildfire paradigm and likely extend beyond the burn zone (16, 21, 22).

Although these cross-sectional data do not establish causality, several converging features strengthen causal plausibility. Internal biomarkers remained elevated months after the fire, exposure gradients were spatially concordant with wildfire impact and environmental contamination, associations were directionally consistent across three independent mixture-modeling approaches, and primary findings persisted in analyses restricted to nonsmokers. In addition, extensive quality assurance and quality control evaluations demonstrated negligible contributions from urine collection, storage, and aliquoting materials, supporting the validity of the measured urinary metal concentrations and minimizing the likelihood that the observed findings reflect analytical artifacts. Together, these features reduce the likelihood that the observed associations are explained by model choice, smoking-related confounding, or measurement bias and support the interpretation that persistent internal metal mixtures represent a clinically relevant component of postdisaster health burden. The results further showed that only a subset of individual metals demonstrated significant associations with pulmonary function outcomes, whereas mixture-based approaches identified a more consistent overall relationship. These findings support the hypothesis that combined exposure to correlated wildfire-related metal mixtures may be more relevant to adverse respiratory health outcomes than single-metal effects considered in isolation.

WUI fires mobilize complex mixtures of metals from vegetation, soils, and the built environment, including treated lumber, electrical systems, vehicles, and household materials (16, 17). Unlike wildland-only fires, these events redistribute structural and industrial contaminants alongside combustion residues, creating multisource exposure environments. The metals most elevated in MauiWES, including antimony, manganese, barium, and arsenic, mirror those identified in postfire ash, soil, and settled dust following the August 2023 Maui wildfires (25). Although Hawaiʻi’s volcanic geology is associated with elevated background levels of some elements, including arsenic and manganese, the magnitude, mixture structure, and spatial heterogeneity observed here exceed what would be expected from undisturbed geological characteristics alone. Elevations were greatest in wildfire-impacted regions and were enriched for metals characteristic of structural combustion, including antimony, cadmium, and copper, suggesting a shift in exposure profiles beyond geogenic baselines and consistent with the combined influence of newly generated combustion residues and mobilized legacy contaminants. Similar evidence from the 2025 Los Angeles urban fires—including elevated airborne lead, airborne hexavalent chromium nanoparticles near cleanup zones, and postfire soil exceedances for lead, cadmium, arsenic, and polycyclic aromatic hydrocarbons (PAHs)—reinforces that WUI disasters generate multicontaminant exposure profiles extending beyond any single sentinel toxicant (26–29).

The persistence of elevated urinary metals up to 18 mo after the disaster suggests sustained or intermittent exposure rather than transient acute uptake. Wildfire debris deposition, soil contamination, and resuspension of fine particulates can maintain exposure pathways long after visible smoke has dissipated (25, 30, 31). Plausible ongoing pathways include inhalation of resuspended particulates, contact with contaminated dust and debris, and environmental ingestion through hand-to-mouth or other indirect exposure routes. This temporal decoupling between the fire event and internalized dose has important implications: Health risk may extend well beyond the acute response window and proximal disaster-affected areas typically emphasized in disaster management. Reliance on short-term air monitoring alone may therefore underestimate prolonged internal toxic burden after WUI disasters, even after ambient concentrations decline.

Spatial patterns further support the interpretation that postdisaster exposure was heterogeneous and extended beyond the immediate burn zone. Mixture–lung function associations were strongest in Lahaina and Kula, two of the regions most directly affected by the wildfire, and the metals most elevated in Lahaina residents—particularly arsenic, antimony, lead, cobalt, and copper—substantially overlapped with those identified in postfire ash and surface dust by the Hawaiʻi Department of Health (HDOH) (32). At the same time, elevations observed in Wailuku, Kahului, and Kihei indicate that wildfire-related exposure operated within a broader environmental context rather than as an isolated burn zone phenomenon. Re-entrained particulates, historically contaminated soils, and nonfire industrial or agricultural sources likely contributed to regional heterogeneity in internal burden. Urinary lead concentrations were only modestly elevated relative to NHANES despite substantial lead content in postfire debris, likely reflecting biomarker kinetics, because urinary metals capture subchronic internalized exposure whereas circulating lead more directly reflects recent blood levels; HDOH screening likewise found that only a small proportion of residents and first responders had blood lead concentrations above the reference range, and levels remained below those associated with overt clinical toxicity (33). Taken together, these regional patterns indicate that the internal exposure footprint of a WUI disaster is shaped by both direct wildfire impact and broader landscape processes.

The Maui wildfires also occurred within a legacy landscape shaped by plantation-era land use, including arsenic-treated materials, agrichemical residues, and persistent soil contamination (34–36). Evidence from HDOH ash characterization (32) and coastal sampling after the fire (37) suggests that the disaster not only generated new metal-rich ash but also mobilized preexisting contaminants. Similar amplification of legacy pollution has been documented after floods, hurricanes, and other debris-disturbing events, where previously stable contaminants become bioavailable (34, 35). These interactions are especially important in communities already facing structural vulnerability. Native Hawaiian and Pacific Islander populations, for example, already experience elevated burdens of asthma, COPD, and cardiovascular disease risk (38–40) and were disproportionately affected by displacement after the Maui wildfires. Situating WUI exposure within this broader socioenvironmental context is therefore essential for understanding how climate-driven disasters can compound preexisting environmental and health inequities.

This study has several strengths, including community-engaged recruitment across wildfire-impacted and comparison regions, standardized spirometry conducted in accordance with ATS/ERS guidelines, and direct biomonitoring of internal metal burden integrated with multimethod mixture modeling. Several limitations warrant consideration. The cross-sectional design precludes causal inference, and the absence of prefire biomarker data limits the ability to quantify within-person change attributable to the disaster. Although NHANES III lung function equations are widely used in clinical practice, they may not fully capture Hawaiʻi’s sociodemographic composition, particularly in populations underrepresented in the original reference sample (41–43). Reliance on spot urine samples may incompletely capture longer-term exposure variability, potentially attenuating effect estimates. Because the cohort was community-engaged rather than population-based, it may not be fully representative of all Maui residents. Mixture modeling prioritizes joint exposure effects and may limit inference regarding the independent contribution of specific components; metals with low or unstable weights should therefore not be interpreted as biologically irrelevant. Urinary metals also do not capture the full spectrum of wildfire-related toxicants, and some metals may serve as proxies for co-occurring unmeasured exposures relevant to respiratory health, including polycyclic aromatic hydrocarbons, volatile organic compounds, and other combustion-related pollutants. Total urinary arsenic may partly reflect dietary organic arsenic exposure and should therefore be interpreted primarily as a component of the broader postdisaster mixture rather than as a fully source-specific marker. Comparisons with NHANES 2011–2012 require cautious interpretation given secular declines in background exposures and Hawaiʻi’s distinct geologic context, although the within-cohort spatial gradients mitigate this concern. At the same time, urinary metals provide a practical measure of internalized exposure in postdisaster settings, although the toxicokinetic properties and biological half-lives of individual metals vary across analytes. The extended recruitment period may limit our ability to attribute all measured exposures exclusively to the initial wildfire event, particularly among participants enrolled later after the disaster. However, the consistency of findings across primary models and sensitivity analyses adjusting for time since the fire suggests that the observed associations were not primarily driven by differences in sample collection timing. Furthermore, extensive quality assurance and quality control analyses demonstrated negligible contamination from urine collection, storage, and aliquoting materials, supporting the validity of the measured urinary metal concentrations. Taken together, the spatial concordance of exposure patterns with wildfire impact zones, agreement with environmental sampling data, robustness across multiple analytic approaches, and strong analytical quality control support the relevance of disaster-related exposure processes as contributors to the observed associations.

The implications of these findings extend beyond the immediate aftermath of wildfire events. Post-WUI disaster response should incorporate extended biomonitoring, targeted respiratory evaluation, and environmental remediation strategies that address both newly generated combustion residues and mobilized legacy contaminants. Expanded chemical surveillance, including persistent organic pollutants and other combustion by-products, may be needed to more fully characterize the cumulative toxic burden. As climate-driven fires increase in frequency and severity, especially in regions with legacy contamination and structural vulnerability, disaster-response frameworks should move beyond short-term air monitoring alone and incorporate mixture-focused biomonitoring and longer-term respiratory surveillance. More broadly, these findings underscore the importance of evaluating cumulative exposure burden rather than relying on single sentinel toxicants. Integrating mixture-focused biomonitoring into disaster preparedness and recovery may be critical to identifying and mitigating persistent respiratory risk after WUI disasters.

## Materials and Methods

### Study Design and Participants.

Data were drawn from the Maui Wildfire Exposure Study (MauiWES), a community-engaged cohort evaluating the health impacts of the August 2023 Maui wildfires. Between January 2024 and February 2025 (6 to 18 mo postdisaster), adults (≥18 y) residing in impacted and adjacent communities were recruited. The final analytic sample comprised 1,400 nonpregnant participants with complete covariate, urinary metal, and spirometry data (44). The study protocol was approved by the University of Hawaiʻi Institutional Review Board, and all participants provided written informed consent.

### Clinical and Environmental Assessments.

Pulmonary function was assessed via spirometry using EasyOne Air devices in accordance with ATS/ERS standards and interpretive guidance (43, 45). Abnormal lung function was defined as values below the LLN using NHANES III reference equations (46). To assess internal metal burden, spot urine samples were collected concurrently and analyzed for a panel of trace metals using an Agilent 8900 inductively coupled plasma triple quadrupole mass spectrometer (ICP-QQQ-MS). Metal concentrations were adjusted for urine specific gravity to account for hydration status.

### Statistical Mixture Modeling.

Associations between cumulative urinary metal mixtures and abnormal pulmonary function were evaluated using three complementary frameworks: dual-index weighted quantile sum (WQS) regression, quantile g-computation (Q-gComp), and Bayesian kernel machine regression (BKMR). We used these complementary approaches because each captures a different aspect of mixture behavior. Dual-index WQS regression estimates the overall association of correlated mixtures while identifying the metals that contribute most strongly in positive and negative directions. Q-gComp allows mixture components to contribute in mixed directions and estimates the joint effect of increasing all exposures simultaneously. BKMR complements these approaches by evaluating potential nonlinearities and the relative importance of individual metals within the mixture. All models were adjusted for age, sex, BMI, and smoking status. Detailed protocols regarding laboratory quality control, spirometry grading, and iterative mixture screening procedures are provided in *SI Appendix*.

## Supplementary Material

Appendix 01 (PDF)

## Data Availability

The statistical code and analytic data used in this study are available at https://doi.org/10.7910/DVN/JI6Y97 (44).
