# Supplementary material for: Climate-amplified wildland–urban interface disasters create persistent internal metal mixtures associated with pulmonary impairment
Source: Proc Natl Acad Sci U S A. 2026 Sep 28;123(40):e2613208123. doi: 10.1073/pnas.2613208123 (PMC13643288; doi:10.1073/pnas.2613208123)
Supplement: Supplementary file 1 [file pnas.2613208123.sapp.pdf]

Supporting Information

Title

Climate-amplified wildland–urban interface disasters create persistent internal metal mixtures associated with pulmonary impairment

Authors

Alika K. Maunakea, PhD[1] , Binh Le, PhD[2,3], Rafael E. de la Hoz, MD, MPH, MS[4], Alison G. Lee, MD MS[5], Zitong Zhang, PhD[4], Martina Kamaka MD[6], Marsha Lowery, ND[7], Lesley Umeda[1], Brennan Yamamoto[1], Kathrin Schilling PhD[8], Ruben Juarez, PhD[2,3,*]

SI Materials and Methods

Study design and participants. MauiWES is an ongoing, community-engaged cohort established to evaluate the biological, psychological, and environmental health impacts of the August 2023 Maui wildfires. Details of the study design and initial baseline findings have been described previously (ref. 23-24 in the main text). Between January 2024 and February 2025 (6–18 months post-disaster), adults aged ≥18 years residing in directly impacted and adjacent communities were recruited through community partners and the Hawaiʻi State Department of Health (HDOH).

A community advisory board guided study recruitment strategies and data governance to uphold principles of Indigenous data sovereignty and equitable participation. Participants completed standardized questionnaires assessing demographics, residential history, wildfire-related exposures, tobacco use, medical history, and psychosocial stressors. Clinical assessments included blood pressure, anthropometry (for body mass index [BMI]), spirometry, and collection of urine, saliva, and blood specimens.

Of 1,837 adults enrolled at baseline, 1,400 participants with complete urinary metal measurements and spirometry data were included in the present analysis. Urine collection and spirometry were performed during the same visit to ensure temporal alignment of exposure and outcome assessment. Exclusion criteria included pregnancy, acute respiratory illness at the time of assessment, and missing key covariates (age, sex, or BMI). At the time of the fires, participants resided primarily in Lahaina (65%), with additional representation from Kula (8.8%), Wailuku/Kahului (15.5%), and Kihei (10.6%).

Urinary metal analysis. Spot urine samples were collected in pre-screened, trace metal-free containers and stored at −80 °C until analysis. Trace element measurements were performed at the Columbia University Trace Metals Core Facility using an Agilent 8900 inductively coupled plasma triple quadrupole mass spectrometer (ICP-QQQ-MS) (1). The urinary panel included 27 metals listed in **Table S1**. Urine aliquots (0.1 mL) were mixed with 50

µL of an internal standard solution (500 µg/L Ga, Y, Rh, Ir) and diluted to 5 mL with a 2% nitric acid and 500 µg/L gold solution. Quality control included procedural blanks, duplicate sample analysis and certified urine reference materials from the Quebec Multi-element External Quality Assessment Scheme (QM-U-Q2313 and QM-U-Q2315), NIST SRM 2668 (Levels 1 and 2), Seronorm Level 1 and ClinChek Level 1. Limits of detection (LOD) were calculated as 3.33 × the standard deviation (SD) of blank measurements and multiplying by dilution factors of 50. Measurements below the LOD were imputed as LOD ÷ √2 (2). Due to a high number of values below the LOD, the metals bismuth, uranium, and palladium were excluded from analysis. To account for hydration status, urinary trace element concentrations were adjusted for urine specific gravity (USG) (3, 4).

Urinary element concentrations (Cnorm) were normalized for hydration status using the Levin-Fahy equation: Cnorm = Cmeasured (SGmedian − 1)/(SGmeasured − 1), where Cmeasured is the measured urine element concentration and SGmeasured is the urine specific gravity. SGmedian describes the median value of the MauiWES cohort. Because metal distributions were right-skewed, USG-corrected metal concentrations were natural logarithm transformed for all regression and mixture analyses. To contextualize exposure levels, MauiWES metal concentrations were compared with the levels found in the 2011–2012 NHANES cycle, the most recent survey period collected to include urinary metals and pulmonary data. The uncorrected concentrations of metals from the MauiWES cohort were collected for direct comparisons with NHANES (5). Fold differences and population percentile shifts were computed to estimate deviation from U.S. background levels found in the NHANES data.

Background metal assessment of urine collection containers and aliquoting vials. To evaluate potential metal contamination from urine collection and aliquoting materials, 100 mL urine collection cups and 2 mL Corning™ polypropylene (PP) cryogenic aliquoting vials were filled with ultrapure deionized water (≥18.2 MΩ·cm) at volumes representative of expected use and aliquoting conditions (**Table S11**). Urine collection cups were filled with 30, 50, or 80 mL, whereas aliquoting vials were filled with 0.5, 1, or 2 mL. These fill volumes were selected to capture differences in liquid-contact surface area, headspace, and sample-to-container volume ratio that may influence background metal release. Urine cups and vials were agitated overnight on a shaker at 25°C. Following incubation, a 0.5 mL aliquot of the leachate from each container or vial was transferred into metal-free 15 mL PP tubes and diluted to a final volume of 5 mL with 2% (v/v) nitric acid containing gold and internal standards, and analyzed using the same procedures applied to urine specimens. Median and standard deviation (SD) concentrations were calculated from replicate measurements (aliquoting vials, n = 12; urine collection containers, n = 9). The contribution of background metals from each material was expressed as a percentage of the median urine concentration measured in the MauiWES cohort. Values reported as <0.01% indicate that the median leachate concentration was below the procedural blank.

Selecting metals for mixture signatures. We used principal component analysis (PCA) to characterize correlation structure and guide dimensionality reduction among measured metals (6). Sixteen metals with the strongest overall contributions to variance were retained for mixture modeling. In addition, arsenic was prespecified for inclusion based on its established toxicological relevance, widespread use in urinary biomonitoring, and documented enrichment in post-disaster ash and soil samples (ref. 32 in main text). This approach balanced data-driven dimensionality reduction with biological plausibility. To estimate mixture effects, we applied weighted quantile sum (WQS) regression (ref. 18 in main text) and quantile g-computation (Q-gComp) (ref. 19 in main text) using a structured iterative screening strategy. In the first two iterations, metals meeting weight thresholds (≥ 0.05 in WQS or ≥ 0.10 in Q-gComp) were retained. In the final iteration, metals were required to meet weight criteria and demonstrate consistent directional influence across at least two spirometry outcomes. This process yielded overall and region-specific metal mixture signatures described below.

Multiple linear regression models. We first evaluated associations between individual urinary metal concentrations and pulmonary function outcomes using multivariable linear regression models. Urinary metal concentrations were natural log-transformed to reduce skewness and modeled as independent variables, while pulmonary function measurements served as dependent variables. The general model specification was:

$$Y_i = \beta_0 + \sum_{1}^{k} \beta_k Z_i + Age_i + Sex_i + Smoking_i + BMIScore_i + \varepsilon_i$$

Where Y_i denotes the pulmonary function measurement for participant i; Z_i represents the log-transformed concentration of metal k for participant i, including urinary arsenic, antimony, cadmium, copper, lead, and calcium. β_0 is the intercept; β_k represents the regression coefficient associated with each metal exposure; and ε_i is the random error term. All models were adjusted for age, sex, smoking status, and body mass index (BMI). Although conventional multivariable regression provides an interpretable framework for evaluating associations between individual exposures and health outcomes, the analysis of environmental mixtures presents several methodological challenges. Specifically, mixtures often comprise numerous correlated components that may exhibit non-linear exposure–response relationships and complex interactions. Under these conditions, traditional regression approaches may yield unstable estimates and may not adequately characterize the joint effects of co-occurring exposures. To address these challenges, we complemented conventional regression analyses with three mixture-modeling approaches designed to estimate the collective effects of metal mixtures on pulmonary function and to capture potential non-linear and non-additive relationships among mixture components, described below.

The Weighted Quantile Sum (WQS) regression approach. The WQS regression model was used to construct a weighted regression coefficient and estimate the association of mixed-metal exposure with the pulmonary outcomes. The basic WQS (ref. 18 in main text) is described as:

$$g(\mu) = \beta_0 + \beta_1 \left(\sum_{i=1}^{c} \omega_i q_i \right) + z'\varphi$$

Where ω_i is the unknown weight for the *i*-th component, β_0 is the intercept, β_1 is the regression coefficient for the weighted quantile sum (constraining its association with the mean to be either non-positive or non-negative), *z* is a vector of confounders, including age, sex, smoking status, and BMI score, and *g* represents any monotonic, differentiable link function as in a generalized linear model, which links the mean (μ) to the predictor variables. The term $\sum_{i=1}^{c} \omega_i q_i$ represents the weighted index for the set of *c* metals of interest. The weights are constrained to sum to 1, $\sum_{i=1}^{c} \omega_i = 1$ and constrained by the limit $0 \leq \omega_i \leq 1$.

The Quantile-based g-Computation (Q-gComp) approach. Q-gComp estimates the joint effect of increasing all mixture components simultaneously while allowing individual exposures to contribute in different directions. Unlike standard WQS regression, it does not require all mixture components to act in the same direction. This makes Q-gComp particularly useful when correlated environmental mixtures include heterogeneous effects across components. We therefore used Q-gComp as a complementary approach to evaluate whether the overall mixture–pulmonary associations were robust to mixed-direction contributions among metals.

The Bayesian Kernel Machine Regression (BKMR) approach. Although WQS and Q-gComp have demonstrated utility for evaluating associations between individual or mixed-metal exposures and health outcomes, these approaches have limited capacity to characterize complex interactions among mixture components when multiple metals are simultaneously present (8). BKMR provides a flexible semiparametric framework for estimating multivariable exposure–response functions without imposing strict assumptions of linearity or additivity (9). By modeling the joint effects of correlated exposures, BKMR can identify potential non-linear exposure–response relationships and non-additive interactions among mixture components, thereby offering important insights into the biological and epidemiological complexity of metal mixtures. As such, BKMR serves as a valuable complement to WQS-based approaches, helping to address limitations associated with summarizing mixtures using a single weighted index. Through visualization of univariate and bivariate exposure–response functions, BKMR enables the assessment of how the association between a given metal and an outcome may vary across different levels of co-occurring metals, providing a powerful framework for investigating interactions within complex environmental mixtures. The basic BKMR model is described as:

$$Y_i = h(z_i) + x_i\beta + \varepsilon_i$$

Where Y_i represents pulmonary function metrics of an individual *i*; *h* is a high-dimensional exposure function; *z* is the vector of urinary metal variables; *x* is a vector of covariates, including age, sex, smoking status, and BMI score; and ε is the residual that follows a normal distribution.

Pulmonary function testing. Spirometry was performed by trained technicians using EasyOne Air devices (ndd Technologies AG, Zürich, Switzerland) in accordance with ATS/ERS spirometry standards (10, ref. 44 in main text). Each participant completed at least three acceptable maneuvers, typically six, and the highest values from reproducible tests were retained. Forced expiratory volume in one second (FEV_1), forced vital capacity (FVC), and forced expiratory flow at 25–75% of FVC ($FEF_{25–75}$) were expressed as percent predicted using NHANES III reference equations (ref. 45 in the main text). FEV_1 and FVC were taken from the best acceptable maneuver and used to calculate the FEV_1/FVC ratio. Abnormal lung function was defined as values below the lower limit of normal (LLN, <5th percentile) according to NHANES III reference equations. Quality review was performed by trained technicians and external reviewers, and sessions with ATS/ERS quality grades A–C were included in analyses.

Covariates. All models were adjusted for age, sex, BMI, and smoking status. Age was treated as a continuous variable and calculated from the participant's reported year of birth. Sex was assessed using the survey item, "What sex was originally listed on your birth certificate?", with response options of male, female, other, or decline to answer. For analysis, sex was dichotomized as male [1] and female [0]. Smoking status was derived from responses to the question, "Do you use tobacco products? If so, how often?", with options including no, yes daily, yes less than monthly, yes monthly, and yes weekly. For modeling, smoking status was dichotomized as current smoker [1], including "yes daily," "yes less than monthly," and "yes monthly" versus [0] non-smoker. BMI was calculated from measured height and weight at study entry and expressed in kg/m^2. Burn-zone classification was assigned by geocoding participants' residential ZIP codes at the time of the fires.

Statistical analysis. To assess regional differences in wildfire-related exposure, we developed a self-reported measure of smoke and ash exposure based on participants' recall of exposure during and immediately after the Maui wildfires. Participants were asked, *"How frequently have you been exposed to wildfire ash, debris, or smoke?"* Response options followed a four-level Likert-style scale: never, rarely, daily, and weekly. This variable was analyzed as an ordinal indicator of exposure frequency for descriptive purposes. To evaluate regional differences in exposure patterns across Maui, we compared the distribution of response categories by region using a chi-square test of independence. Participants with missing responses were retained in descriptive summaries but excluded from statistical comparisons. Urinary metal distributions were summarized as geometric medians and interquartile ranges (IQRs). Differences between MauiWES and NHANES were assessed using two-sample t-tests for log-transformed concentrations and Wilcoxon rank-sum tests for medians. Associations between individual metals and abnormal lung function were evaluated using generalized linear models (GLMs) with a logit link to estimate odds ratios (ORs) and 95% confidence intervals (CIs) per 1-SD increase in log-transformed concentration. Urinary metal mixture effects were evaluated using three frameworks, the first of which we

considered our primary analysis followed by two secondary analyses to confirm the mixture: (1) Dual-directional WQS using bootstrap resampling to estimate mixture effects and component weights (ref. 18 in main text); (2) Q-gComp, permitting mixed-direction contributions (ref. 19 in main text); and (3) BKMR, to model non-linear and interactive effects and compute posterior inclusion probabilities (PIPs) (ref. 20 in main text). Analyses were conducted in R version 4.4.2 using the *gWQS*, *qgcomp*, and *bkmr* packages. Regional heterogeneity was assessed by fitting stratified models. Sensitivity analyses were restricted to non-smokers. Two-sided p values < 0.05 were considered statistically significant.

Ethical approval. The study protocol was approved by the University of Hawai‘i Institutional Review Board (Protocol # 2021-00989). All participants provided written informed consent. The research adhered to principles of community partnership, equity, and transparency as described previously (ref. 23, 24 in main text).

Data deposition. The statistical code and analytic data used in this study are available at https://doi.org/10.7910/DVN/JI6Y97.

Antimony	Arsenic	Barium	Bismuth	Bromine	Cadmium	Calcium
Cesium	Chromium	Cobalt	Copper	Iron	Lead	Lithium
Magnesium	Manganese	Molybdenum	Nickel	Palladium	Selenium	Strontium
Thallium	Tin	Tungsten	Uranium	Vanadium	Zinc	(Gravity)

Table S1. Urinary metals panel measured in the Maui Wildfire Exposure Study (MauiWES) cohort. The panel includes 27 urinary metals and trace elements quantified to assess wildfire-related environmental exposures and internal toxicant burden among study participants. Urinary specific gravity was measured for normalization of metal concentrations.

Characteristics	Overall (N = 1,803)	Kihei (N = 192)	Kula (N = 159)	Lahaina (N = 1,172)	Wailuku/Kahului (N = 280)
**Sex**					
Female	1,028 (60.94%)	120 (64.86%)	97 (61.01%)	669 (59.89%)	142 (62.83%)
Male	659 (39.06%)	65 (35.14%)	62 (38.99%)	448 (40.11%)	84 (37.17%)
Missing/Not reported	116	7	0	55	54
**Age**	47.0 (36.0-60.0)	45.0 (35.0-58.5)	44.0 (31.0-56.0)	49.0 (38.0-61.0)	41.0 (31.5-54.0)
Missing/Not reported	60	4	0	32	24
**Race/Ethnic group**					
Native Hawaiian or Other Pacific Islander	338 (20.23%)	17 (9.29%)	40 (25.32%)	202 (18.25%)	79 (35.43%)
Non-Filipino Asian	144 (8.62%)	12 (6.56%)	20 (12.66%)	91 (8.22%)	21 (9.42%)
Filipino	315 (18.85%)	42 (22.95%)	3 (1.90%)	214 (19.33%)	56 (25.11%)
Hispanic/Latino	292 (17.47%)	26 (14.21%)	13 (8.23%)	239 (21.59%)	14 (6.28%)
White	487 (29.14%)	77 (42.08%)	74 (46.84%)	293 (26.47%)	43 (19.28%)
Other	95 (5.69%)	9 (4.92%)	8 (5.06%)	68 (6.14%)	10 (4.48%)
Missing/Not reported	132	9	1	65	57
**Smoke/ash exposure**					
Never	225 (13.63%)	35 (19.23%)	21 (13.46%)	95 (8.65%)	74 (34.42%)
Rarely	819 (49.61%)	108 (59.34%)	73 (46.79%)	550 (50.09%)	88 (40.93%)
Weekly	303 (18.35%)	22 (12.09%)	28 (17.95%)	219 (19.95%)	34 (15.81%)
Daily	304 (18.41%)	17 (9.34%)	34 (21.79%)	234 (21.31%)	19 (8.84%)
Missing/Not reported	152	10	3	74	65
**Smoking status**					
No	1,377 (83.25%)	165 (91.67%)	130 (82.28%)	909 (82.79%)	173 (79.36%)
Yes	277	15	28	189	45

	(16.75%)	(8.33%)	(17.72%)	(17.21%)	(20.64%)
Missing/Not reported	149	12	1	74	62
**Months from fire to data collection**	12.19 (6.54-15.54)	12.32 (11.89-13.04)	11.89 (6.34-13.04)	10.71 (6.31-16.03)	13.04 (12.35-16.00)
**BMI**	27.88 (24.13-32.68)	27.57 (23.52-31.68)	27.97 (23.81-32.29)	27.6 (23.97-32.44)	29.54 (25.47-34.66)
Missing/Not reported	49	3	2	29	15

Table S2. Demographic and clinical characteristics of the analytic sample overall and stratified by geographic region. Participants residing outside Maui County ZIP codes were excluded from all analyses. Categorical variables are presented as counts and percentages, and continuous variables are presented as median and interquartile range (IQR), as appropriate.

	Overall (N = 1,759)	Kihei (N = 188)	Kula (N = 158)	Lahaina (N = 1,140)	Wailuku / Kahului (N = 273)	*p*-value
**All Spirometry Sessions**						
FVC < LLN						0.002
No	1,313(77.01%)	156 (84.32%)	133 (84.71%)	832 (75.77%)	192 (72.45%)	
Yes	392 (22.99%)	29 (15.68%)	24 (15.29%)	266 (24.23%)	73 (27.55%)	
Missing/Not reported	54	3	1	42	8	
FEV_1 < LLN						0.001
No	1,184 (69.44%)	140 (75.68%)	124 (78.98%)	753 (68.58%)	167 (63.02%)	
Yes	521 (30.56%)	45 (24.32%)	33 (21.02%)	345 (31.42%)	98 (36.98%)	
Missing/Not reported	54	3	1	42	8	
FEV_1/FVC < LLN						0.2
No	1,391 (81.58%)	148 (80.00%)	138 (87.90%)	890 (81.06%)	215 (81.13%)	
Yes	314 (18.42%)	37 (20.00%)	19 (12.10%)	208 (18.94%)	50 (18.87%)	
Missing/Not reported	54	3	1	42	8	
FEF_{25-75} < LLN						0.055
No	1,344 (78.83%)	150 (81.08%)	133 (84.71%)	865 (78.78%)	196 (73.96%)	
Yes	361 (21.17%)	35 (18.92%)	24 (15.29%)	233 (21.22%)	69 (26.04%)	
Missing/Not reported	54	3	1	42	8	
**Only A-C Quality Spirometry Sessions**						
FVC < LLN						0.2
No	750 (85.13%)	104 (91.23%)	77 (87.50%)	461 (83.97%)	108 (83.08%)	
Yes	131 (14.87%)	10 (8.77%)	11 (12.50%)	88 (16.03%)	22 (16.92%)	
FEV_1 < LLN						0.058
No	774 (79.96%)	100 (85.47%)	79 (84.04%)	486 (79.93%)	109 (73.15%)	
Yes	194 (20.04%)	17 (14.53%)	15 (15.96%)	122 (20.07%)	40 (26.85%)	
FEV_1/FVC < LLN						0.6
No	681 (91.78%)	90 (91.84%)	74 (94.87%)	420 (90.91%)	97 (93.27%)	
Yes	61 (8.22%)	8 (8.16%)	4 (5.13%)	42 (9.09%)	7 (6.73%)	
FEF_{25-75} < LLN						0.2
No	667 (89.89%)	89 (90.82%)	75 (96.15%)	410 (88.74%)	93 (89.42%)	
Yes	75 (10.11%)	9 (9.18%)	3 (3.85%)	52 (11.26%)	11 (10.58%)	

Table S3. Comparison of abnormal pulmonary function by region in the MauiWES (2024–2025) cohort. Values are presented as N (%). Chi-square tests were used to compare distributions across regions. In the full set of interpretable spirometry sessions (N = 1,759), regional sample sizes were Kihei (N = 188), Kula (N = 158), Lahaina (N = 1,140), and Wailuku/Kahului (N = 273). For analyses restricted to spirometry tests meeting ATS/ERS quality grades A–C, the number of available tests varied by outcome. For FVC < LLN, the A–C subsample consisted of Kihei (N = 114), Kula (N = 88), Lahaina (N = 549), and Wailuku/Kahului (N = 130). For FEV_1 < LLN, the A–C subsample included Kihei (N = 117), Kula (N = 94), Lahaina (N = 608), and Wailuku/Kahului (N = 149). For FEV_1/FVC < LLN or $FEF_{25–75}$ < LLN, the corresponding A–C subsample sizes were Kihei (N = 98), Kula (N = 78), Lahaina (N = 462), and Wailuku/Kahului (N = 104). The reported *p*-values indicate the statistical significance of regional differences for each pulmonary outcome.

Metal	Kihei (N = 182)	Kula (N = 139)	Lahaina (N = 1,041)	Wailuku/Kahului (N = 256)	*p*-value
Lithium	23.14 (17.32-34.78)	21.84 (17.13-33.16)	21.39 (16.23-31.00)	19.44 (14.26-25.18)	<0.001
Missing/Not reported	0	0	3	0	
Magnesium	67,829.86 (43,620.47-88,711.21)	59,386.84 (37,814.98-85,592.02)	58,319.48 (40,219.28-81,510.76)	56,673.70 (36,439.10-81,983.76)	0.044
Missing/Not reported	0	0	3	0	
Calcium	118,310.65 (67,080.07-167,152.20)	109,210.15 (66,161.07-190,713.25)	103,083.97 (59,429.97-162,049.59)	102,239.48 (62,561.75-158,188.42)	0.15
Missing/Not reported	0	0	3	0	
Vanadium	0.30 (0.21-0.44)	0.24 (0.16-0.34)	0.24 (0.17-0.38)	0.30 (0.23-0.45)	<0.001
Missing/Not reported	0	0	3	0	
Chromium	1.54 (1.19-2.49)	1.66 (1.27-3.01)	1.64 (1.27-2.70)	1.80 (1.22-3.21)	0.14
Missing/Not reported	0	0	3	0	
Manganese	0.56 (0.37-0.85)	0.55 (0.39-0.87)	0.53 (0.38-0.82)	0.56 (0.39-0.86)	0.6
Missing/Not reported	0	0	3	0	
Iron	16.35 (12.38- 20.58)	15.57 (12.46- 21.77)	16.38 (12.76- 22.09)	18.46 (12.89-24.37)	0.031
Missing/Not reported	0	0	3	0	
Cobalt	0.43 (0.31-0.69)	0.44 (0.35-0.71)	0.39 (0.30-0.58)	0.40 (0.30-0.63)	0.025
Missing/Not reported	0	0	3	0	
Nickel	3.89 (2.60-5.60)	3.28 (2.16-5.07)	2.37 (1.72-3.58)	3.28 (2.24-4.74)	<0.001
Missing/Not reported	0	0	3	0	
Copper	11.91 (8.46-17.13)	10.42 (7.84-14.78)	10.64 (7.87-15.17)	12.53 (8.77-19.89)	<0.001
Missing/Not reported	0	0	3	0	
Zinc	698.68 (495.60- 1,010.16)	645.62 (482.81- 982.04)	692.44 (476.61- 990.15)	766.14 (514.51- 1,021.65)	0.3
Missing/Not reported	0	0	3	0	
Arsenic	19.69 (8.16- 46.52)	17.82 (7.76- 36.96)	22.34 (9.83-55.40)	22.54 (10.58-65.05)	0.010
Missing/Not reported	0	0	3	0	
Bromine	3,103.33 (2,290.69-4,179.76)	2,724.29 (2,126.81-3,478.80)	3,009.71 (2,142.28-3,979.64)	2,907.78 (1,985.16-3,816.93)	0.015
Missing/Not reported	0	0	3	0	
Selenium	45.23 (32.67- 62.61)	50.69 (35.22- 65.20)	48.47 (35.19- 66.72)	48.08 (33.92-69.26)	0.6
Missing/Not reported	0	0	3	0	
Strontium	158.72 (103.62- 243.66)	138.28 (92.18- 207.91)	137.92 (87.52- 205.78)	132.50 (95.02- 192.33)	0.046
Missing/Not reported	0	0	3	0	

Molybdenum	57.68 (32.60- 91.15)	51.88 (36.79-81.22)	49.70 (31.99- 74.12)	59.43 (35.23-93.73)	0.003
Missing/Not reported	0	0	3	0	
Cadmium	0.29 (0.16-0.49)	0.22 (0.14-0.36)	0.29 (0.16-0.55)	0.25 (0.14-0.51)	0.019
Missing/Not reported	0	0	3	0	
Tin	0.69 (0.34-1.76)	0.50 (0.28-0.94)	0.69 (0.34-1.55)	0.71 (0.37-1.54)	0.013
Missing/Not reported	0	0	3	0	
Antimony	2.12 (1.63-2.85)	2.15 (1.69-3.35)	2.10 (1.63-3.12)	2.10 (1.66-3.30)	0.8
Missing/Not reported	0	0	3	0	
Cesium	6.71 (5.16-8.78)	7.01 (5.12-8.53)	6.30 (4.87-8.10)	5.90 (4.73-7.83)	0.003
Missing/Not reported	0	0	3	0	
Barium	3.16 (2.14-4.87)	3.52 (2.52-5.06)	3.04 (1.99-4.58)	3.25 (2.32-4.77)	0.010
Missing/Not reported	0	0	3	0	
Tungsten	0.23 (0.15-0.36)	0.23 (0.15-0.36)	0.21 (0.14-0.32)	0.22 (0.14-0.33)	0.2
Missing/Not reported	0	0	3	0	
Thallium	0.19 (0.13-0.26)	0.20 (0.14-0.31)	0.20 (0.14-0.29)	0.17 (0.12-0.22)	<0.001
Missing/Not reported	0	0	3	0	
Lead	0.49 (0.35-0.77)	0.47 (0.30-0.64)	0.46 (0.31-0.68)	0.46 (0.30-0.83)	0.095
Missing/Not reported	0	0	3	0	

Table S4. **Comparison of urinary metal concentrations by geographic region in the MauiWES cohort (N = 1,618).** Values are presented as median and interquartile range (IQR). Regional differences were evaluated using the Kruskal–Wallis rank-sum test. Sample sizes were Kihei (N = 182), Kula (N = 139), Lahaina (N = 1,041), and Wailuku/Kahului (N = 256). Reported concentrations represent urinary metal values corrected for urine specific gravity to account for variation in hydration and urine dilution.

Variable	Mean (95% CI)	50th Percentile (95% CI)	75th Percentile (95% CI)	90th Percentile (95% CI)	95th Percentile (95% CI)
Lithium	39.29 [29.69 - 53.11]	21.14 [20.68 - 21.64]	30.58 [29.34 - 32.01]	46.64 [44.39 - 50.66]	63.77 [57.69 - 68.71]
Magnesium	65339 [63512 - 67171]	59269 [57100 - 61208]	82990.59 [80712 - 85517]	110866 [105231 - 115580]	134288 [126804 - 141548]
Calcium	121445 [117162 - 125513]	104768 [100579 - 109644]	163505 [157271 - 169931]	231248 [222334 - 241606]	274418 [264728 - 293575]
Vanadium	0.74 [0.45 - 1.15]	0.26 [0.25 - 0.27]	0.39 [0.38 - 0.41]	0.62 [0.58 - 0.67]	0.85 [0.77 - 0.96]
Chromium	2.31 [2.23 - 2.40]	1.64 [1.59 - 1.72]	2.78 [2.63 - 2.96]	4.13 [3.89 - 4.36]	5.59 [5.23 - 6.18]
Manganese	0.79 [0.74 - 0.87]	0.54 [0.52 - 0.56]	0.83 [0.79 - 0.87]	1.33 [1.26 - 1.42]	1.77 [1.63 - 1.96]
Iron	24.62 [21.16 - 28.93]	16.51 [16.17 - 16.96]	22.28 [21.54 - 23.12]	32.94 [30.67 - 34.79]	44.23 [40.19 - 49.57]
Cobalt	0.84 [0.60 - 1.28]	0.40 [0.39 - 0.41]	0.60 [0.57 - 0.65]	1.19 [1.09 - 1.31]	1.69 [1.59 - 1.85]
Nickel	3.39 [3.25 - 3.55]	2.73 [2.64 - 2.84]	4.21 [4.00 - 4.35]	5.89 [5.62 - 6.12]	7.19 [6.77 - 7.79]
Copper	16.48 [15.18 - 18.18]	11.01 [10.62 - 11.34]	15.93 [15.27 - 16.79]	24.99 [23.44 - 27.7]	37.98 [34.56 - 42.73]
Zinc	803.81 [779.46 - 828.77]	697.80 [673.44 - 715.06]	996.22 [964.25 - 1020.63]	1398.51 [1310.66 - 1448.23]	1679.44 [1592.08 - 1757.82]
Arsenic	59.76 [51.94 - 69.28]	21.74 [19.63 - 23.78]	53.52 [48.91 - 57.93]	117.8 [106.72 - 132.43]	207.84 [180.17 - 245.96]
Bromine	3171.97 [3094.14 - 3259.15]	2962.28 [2908.29 - 3040.07]	3934.92 [3832.08 - 4028.64]	5056.53 [4841.29 - 5213.53]	5778.99 [5602.56 - 6026.41]
Selenium	54.24 [52.71 - 55.98]	48.16 [46.98 - 49.54]	66.66 [64.47 - 68.58]	86.20 [83.24 - 90.38]	104.17 [99.31 - 108.99]
Strontium	164.01 [156.53 -171.95]	139.06 [134.69 - 144.68]	204.42 [195.95 - 210.98]	279.77 [267.48 - 291.09]	340.86 [322.41 - 355.62]
Molybdenum	64.47 [61.86 - 66.96]	51.68 [49.35 - 53.76]	78.90 [75.42 - 83.13]	119.39 [110.85 - 127.76]	155.59 [141.29 - 168.97]
Cadmium	0.41 [0.39 - 0.43]	0.28 [0.26 - 0.29]	0.52 [0.49 - 0.55]	0.84 [0.80 - 0.90]	1.16 [1.04 - 1.29]
Tin	1.79 [1.57 - 2.06]	0.68 [0.64 - 0.73]	1.51 [1.36 - 1.66]	3.48 [3.02 - 3.94]	6.51 [5.65 - 7.64]
Antimony	2.75 [2.66 - 2.83]	2.11 [2.04 - 2.18]	3.12 [2.95 - 3.29]	5.09 [4.76 - 5.33]	6.54 [6.25 - 7.01]
Cesium	6.83 [6.68 - 6.99]	6.35 [6.24 - 6.49]	8.16 [7.96 - 8.32]	10.32 [9.99 - 10.79]	12.08 [11.61 - 12.65]
Barium	3.97 [3.77 - 4.18]	3.13 [3.03 - 3.23]	4.68 [4.51 - 4.86]	6.65 [6.45 - 6.99]	8.31 [7.79 - 9.04]
Tungsten	0.29 [0.28 - 0.31]	0.22 [0.21 - 0.22]	0.33 [0.32 - 0.35]	0.52 [0.48 - 0.55]	0.69 [0.63 - 0.75]
Thallium	0.23 [0.22 - 0.24]	0.19 [0.19 - 0.19]	0.27 [0.26 - 0.29]	0.37 [0.36 - 0.39]	0.48 [0.44 - 0.52]
Lead	0.72	0.46	0.69	1.16	1.79

	[0.66 - 0.79]	[0.45 - 0.48]	[0.66 - 0.74]	[1.08 - 1.25]	[1.57 - 1.99]
Age	47.98<br>[47.24 - 48.81]	47<br>[46 - 48]	60<br>[59 - 61]	69<br>[68 - 70]	74<br>[73 - 75]
BMI score	32.49<br>[28.69 - 39.92]	27.81<br>[27.47 - 28.19]	32.64<br>[32.16 - 33.26]	37.74<br>[36.83 - 38.57]	41.39<br>[40.66 - 42.57]
Months since the fire	11.12<br>[10.90 - 11.34]	11.89<br>[11.86 - 11.89]	15.11<br>[15.08 - 15.51]	17.55<br>[17.43 - 17.58]	18.04<br>[18.04 - 18.04]

Table S5. Mean and percentile distribution of metal concentrations and other variables (mean, percentiles, and their 95% CI). The table depicts the mean, 50th percentile, 75th percentile, 90th percentile, and 95th percentile of metal concentrations (in µg/L) and continuous variables, including age, BMI, and months since the fire. Variables were derived from the urinary metals dataset (N = 1,618).

Metals	NHANES (2011-2012)	NHANES (2017-2018)	MauiWES (2024-2025)
Barium	0.1	0.06	0.056
Cadmium	0.056	0.036	0.017
Cobalt	0.048	0.023	0.012
Cesium	0.12	0.086	0.011
Molybdenum	0.99	0.8	0.718
Manganese	0.08	0.13	0.068
Lead	0.08	0.03	0.039
Antimony	0.041	0.022	0.035
Tin	0.22	0.09	0.059
Strontium	2.5	n/a	0.2
Thallium	0.02	0.018	0.015
Tungsten	0.026	0.018	0.043
Arsenic	1.25	0.23	0.09

Table S6. Comparison of urinary lower limits of detection (LoDs; µg/L) for matched metals between MauiWES (2024–2025), NHANES (2017-2018), and NHANES (2011–2012). Values represent analytical detection limits for urinary metal concentration. Values in NHANES 2017–2018 were generally lower than those in NHANES 2011–2012, reflecting improvements in analytical sensitivity over time. Strontium is not available (n/a) in the NHANES (2017-2018). Overall, the MauiWES analytical platform achieved lower limits of detection for many metals than NHANES, supporting greater sensitivity for low-level exposure assessment.

Variable	FVC < LLN	FEV_1 < LLN	FEV_1/FVC < LLN	FEF_{25-75} < LLN
Lithium	0.75 [0.46-1.21]	0.60** [0.41-0.88]	0.67 [0.34-1.31]	0.73 [0.42-1.29]
Magnesium	0.96 [0.60-1.52]	0.98 [0.65-1.49]	1.18 [0.59-2.31]	0.82 [0.47-1.45]
Calcium	1.06 [0.58-1.95]	0.83 [0.49-1.37]	0.67 [0.27-1.69]	0.50 [0.22-1.17]
Vanadium	1.04 [0.71-1.54]	1.08 [0.82-1.41]	1.19 [0.79-1.81]	0.94 [0.63-1.40]
Chromium	0.81 [0.47-1.38]	0.83 [0.52-1.32]	1.14 [0.49-2.64]	0.80 [0.33-1.93]
Manganese	1.13 [0.73-1.74]	1.09 [0.73-1.62]	0.66 [0.33-1.27]	1.24 [0.66-2.31]
Iron	1.46+ [0.97-2.18]	1.31 [0.95-1.79]	1.10 [0.72-1.69]	1.39 [0.89-2.19]
Cobalt	1.10 [0.74-1.63]	1.00 [0.71-1.42]	1.38 [0.86-2.22]	0.63+ [0.36-1.08]
Nickel	0.80 [0.46-1.41]	0.82 [0.53-1.28]	0.79 [0.36-1.73]	0.89 [0.43-1.84]
Copper	1.16 [0.73-1.83]	1.25 [0.83-1.87]	0.67 [0.29-1.53]	1.04 [0.53-2.06]
Zinc	0.93 [0.55-1.58]	0.91 [0.59-1.39]	0.93 [0.49-1.75]	1.21 [0.69-2.09]
Arsenic	1.32** [1.08-1.63]	1.14 [0.96-1.35]	0.75+ [0.56-1.01]	1.05 [0.81-1.36]
Bromine	1.09 [0.67-1.75]	0.76 [0.49-1.18]	1.36 [0.60-3.06]	0.75 [0.34-1.68]
Selenium	0.95 [0.52-1.74]	0.89 [0.53-1.47]	1.22 [0.52-2.87]	1.31 [0.55-3.09]
Strontium	0.77 [0.35-1.66]	1.57 [0.88-2.80]	1.45 [0.49-4.25]	2.63* [1.01-6.89]
Molybdenum	1.21 [0.83-1.78]	1.26 [0.92-1.73]	1.12 [0.66-1.91]	0.83 [0.49-1.39]
Cadmium	1.37 [0.93-2.00]	1.33+ [0.97-1.82]	1.00 [0.58-1.73]	2.03* [1.18-3.49]
Tin	1.13 [0.92-1.38]	1.16+ [0.98-1.37]	0.94 [0.71-1.25]	1.11 [0.86-1.44]
Antimony	0.71 [0.29-1.69]	1.17 [0.57-2.39]	1.15 [0.35-3.78]	1.31 [0.41-4.22]
Cesium	0.61 [0.29-1.26]	0.83 [0.44-1.55]	0.45 [0.13-1.54]	0.44 [0.15-1.29]
Barium	1.09 [0.62-1.89]	0.89 [0.57-1.40]	0.96 [0.44-2.08]	0.92 [0.43-1.98]
Tungsten	1.33 [0.93-1.90]	1.03 [0.76-1.39]	1.19 [0.71- 2.03]	1.20 [0.69-2.07]
Thallium	0.68+ [0.43-1.07]	0.92 [0.65-1.30]	1.46 [0.78-2.73]	1.04 [0.62-1.74]
Lead	0.77 [0.51-1.18]	0.85 [0.63-1.17]	2.52*** [1.47-4.31]	1.18 [0.69-2.01]
Age	1.00 [0.99-1.02]	0.99 [0.98-1.01]	1.01 [0.98-1.04]	0.94*** [0.92-0.97]

Sex	1.15<br>[0.67-1.98]	1.24<br>[0.78-1.99]	1.03<br>[0.48-2.22]	0.68<br>[0.31-1.53]
Smoking status	0.61<br>[0.31-1.17]	0.77<br>[0.46-1.31]	1.38<br>[0.59-3.25]	0.91<br>[0.41-2.01]
BMI	1.00***<br>[1.00-1.00]	1.00***<br>[1.00-1.00]	0.95<br>[0.88-1.03]	0.95+<br>[0.89-1.01]

Table S7. Associations between individual urinary concentrations of 24 metals and abnormal pulmonary function estimated using generalized linear models. Each cell reports the odds ratio (OR) and 95% confidence interval (CI) for the association between a single metal and the likelihood of spirometry values falling below the LLN for FEV_1, FVC, FEV_1/FVC, and $FEF_{25–75}$. The models were adjusted for age, sex, smoking status, and BMI, and included spirometry meeting ATS/ERS quality grades A–C. Sample sizes varied by outcome: FEV_1 < LLN (N = 790), FVC < LLN (N = 737), and FEV_1/FVC < LLN and $FEF_{25–75}$ < LLN (N = 624). Statistical significance is indicated as follows: + $p < 0.10$; * $p < 0.05$; ** $p < 0.01$; *** $p < 0.001$.

	Lahaina/Kula				Wailuku/Kahului/Kihei			
	FVC < LLN	FEV_1 < LLN	FEV_1/FVC < LLN	FEF_{25-75} < LLN	FVC < LLN	FEV_1 < LLN	FEV_1/FVC < LLN	FEF_{25-75} < LLN
**WQS**	1.47 [0.91-2.37]	1.32* [1.03-1.69]	1.55* [1.09-2.18]	1.72*** [1.26-2.33]	2.52** [1.39-4.55]	1.98+ [0.99-3.94]	1.51 [0.57-4.02]	2.7*** [1.73-4.2]
Age	1.01 [1-1.03]	1 [0.99-1.01]	1.01 [0.99-1.02]	0.96*** [0.95-0.98]	0.98 [0.95-1.02]	1 [0.96-1.03]	1.06* [1.01-1.1]	0.94+ [0.88-1.01]
Sex	1.06 [0.77-1.46]	1.18 [0.84-1.65]	0.6* [0.39-0.93]	0.77 [0.3-1.95]	1.41 [0.57-3.51]	1.02 [0.37-2.85]	1.38 [0.31-6.2]	0.02 [0-56932]
Smoking status	0.88 [0.6-1.3]	0.88 [0.58-1.35]	2.15** [1.25-3.7]	1.07 [0.47-2.46]	0 [0-21702]	1 [0.94-1.06]	0*** [0-0]	0*** [0-0]
BMI	1.01 [0.99-1.02]	0.99 [0.96-1.01]	0.95* [0.91-1]	0.91*** [0.89-0.93]	1.04*** [1.02-1.05]	1.02 [0.24-4.22]	1.07 [0.98-1.16]	1.03 [0.95-1.13]
**Q-gComp**	1.4 [0.9-2.18]	1.34+ [0.98-1.84]	1.25 [0.58-2.67]	1.89* [1.06-3.36]	1.27 [0.45-3.6]	1.62 [0.88-3]	2.05 [0.29-14.41]	3.33* [1.27-8.72]
Age	1.01 [1-1.03]	1 [0.98-1.01]	1 [0.97-1.03]	0.95*** [0.93-0.98]	0.97+ [0.93-1]	0.98 [0.95-1.01]	1.06+ [1-1.13]	0.94* [0.89-0.99]
Sex	1.16 [0.67-2]	1.21 [0.76-1.93]	1.13 [0.5-2.55]	0.79 [0.37-1.68]	1.71 [0.56-5.24]	1.2 [0.5-2.87]	1.18 [0.28-4.95]	0.39 [0.07-2.35]
Smoking status	0.72 [0.36-1.43]	0.85 [0.48-1.49]	1.62 [0.68-3.9]	1.26 [0.56-2.81]	0.17 [0.02-1.51]	0.38 [0.1-1.48]	0 [0-1]	0 [0-1]
BMI	1 [1-1.01]	1 [1-1]	0.92* [0.85-0.98]	0.92** [0.87-0.98]	1.03 [0.96-1.09]	1.01 [0.96-1.07]	1.06 [0.96-1.18]	1.06 [0.98-1.15]

Table S8. Associations between region-specific metal mixtures and abnormal pulmonary function across two geographic strata: Lahaina/Kula and Wailuku/Kahului/Kihei. Mixture effects were estimated using standard Weighted Quantile Sum (WQS) and Quantile g-computation (Q-gComp) with 500 iterations of bootstrapping, adjusting for age, sex, smoking status, and body mass index. Region-specific metal signatures were derived from iterative mixture selection procedures: the Lahaina/Kula signature comprised arsenic, copper, cadmium, nickel, antimony, and vanadium; the Wailuku/Kahului/Kihei signature comprised arsenic, copper, cadmium, selenium, tungsten, iron, and thallium. Odds ratios (ORs) and 95% confidence intervals (CIs) are reported for each spirometry outcome, including FVC < LLN, FEV_1 < LLN, FEV_1/FVC < LLN, and $FEF_{25–75}$ < LLN. Sample sizes varied across outcomes: in Lahaina/Kula, N = 537 for FVC < LLN, N = 587 for FEV_1 < LLN, and N = 460 for FEV_1/FVC < LLN and $FEF_{25–75}$ < LLN; in Wailuku/Kahului/Kihei, N = 200 for FVC < LLN, N = 203 for FEV_1 < LLN, and N = 164 for FEV_1/FVC < LLN and $FEF_{25–75}$ < LLN. Statistical significance is indicated as follows: + $p < 0.10$; * $p < 0.05$; ** $p < 0.01$; *** $p < 0.001$. Some covariate estimates, particularly for smoking status and sex (in the $FEF_{25–75}$ < LLN outcome) in sparse strata, were unstable because of small sample size and low variability and should not be interpreted.

	Lahaina/Kula				Wailuku/Kahului/Kihei			
	FVC < LLN	FEV_1 < LLN	FEV_1/FVC < LLN	FEF_{25-75} < LLN	FVC < LLN	FEV_1 < LLN	FEV_1/FVC < LLN	FEF_{25-75} < LLN
**WQS**	1.58 [0.91-2.73]	1.55+ [0.98-2.46]	1.41 [0.78-2.57]	1.58+ [0.96-2.58]	3.67** [1.45-9.28]	2.02* [1.01-4.06]	3.2 [0.57-7.92]	7.75* [1.48-40.72]
Age	1.01 [0.99-1.03]	0.99 [0.98-1.01]	1.01 [0.98-1.04]	0.95*** [0.93-0.98]	0.96 [0.92-1]	1.00 [0.97-1.03]	1.07 [0.99-1.16]	0.94 [0.87-1.01]
Sex	1.47 [0.77-2.79]	1.41 [0.81-2.46]	1.08 [0.41-2.84]	0.52 [0.23-1.19]	0.65 [0.18-2.33]	1.03 [0.37-2.82]	0.9 [0.13-6.09]	0.24 [0.02-2.62]
BMI	1.01 [0.96-1.05]	0.99 [0.95-1.03]	0.94 [0.87-1.02]	0.93* [0.87-0.99]	1.03 [0.96-1.11]	1.00 [0.94-1.06]	1.07 [0.95-1.21]	1.05 [0.96-1.15]
**Q-gComp**	1.39 [0.89-2.15]	1.33 [0.96-1.84]	1.27 [0.57-2.85]	1.89* [1.09-3.26]	1.32 [0.49-3.56]	1.65 [0.88-3.09]	2.05 [0.34-12.31]	3.39* [1.22-9.38]
Age	1.01 [0.99-1.03]	0.99 [0.98-1.01]	1 [0.97-1.03]	0.95*** [0.93-0.98]	0.97 [0.93-1.01]	0.99 [0.96-1.02]	0.99 [0.96-1.02]	0.95* [0.89-0.99]
Sex	1.12 [0.65-1.89]	1.18 [0.74-1.86]	1.24 [0.56-2.74]	0.82 [0.39-1.72]	1.51 [0.49-4.58]	1.11 [0.47-2.62]	1.11 [0.47-2.62]	0.37 [0.06-2.16]
BMI	1.00 [0.99-1.01]	1.00 [0.99-1.00]	0.92* [0.85-0.98]	0.92** [0.87-0.98]	1.03 [0.97-1.1]	1.02 [0.96-1.07]	1.02 [0.96-1.07]	1.07 [0.98-1.15]

Table S9. Associations between region-specific metal mixtures and abnormal pulmonary function across two geographic strata: Lahaina/Kula and Wailuku/Kahului/Kihei without controlling for smoking. Mixture effects were estimated using standard Weighted Quantile Sum (WQS) and Quantile g-computation (Q-gComp) with 500 iterations of bootstrapping, adjusting for age, sex, and body mass index. Region-specific metal signatures were derived from iterative mixture selection procedures: the Lahaina/Kula signature comprised arsenic, copper, cadmium, nickel, antimony, and vanadium; the Wailuku/Kahului/Kihei signature comprised arsenic, copper, cadmium, selenium, tungsten, iron, and thallium. Odds ratios (ORs) and 95% confidence intervals (CIs) are reported for each spirometry outcome, including FVC < LLN, FEV_1 < LLN, FEV_1/FVC < LLN, and $FEF_{25–75}$ < LLN. Sample sizes varied across outcomes: in Lahaina/Kula, N = 537 for FVC < LLN, N = 587 for FEV_1 < LLN, and N = 460 for FEV_1/FVC < LLN and $FEF_{25–75}$ < LLN; in Wailuku/Kahului/Kihei, N = 200 for FVC < LLN, N = 203 for FEV_1 < LLN, and N = 164 for FEV_1/FVC < LLN and $FEF_{25–75}$ < LLN. Statistical significance is indicated as follows: + $p < 0.10$; * $p < 0.05$; ** $p < 0.01$; *** $p < 0.001$.

Element (µg/L)	Seronorm L1 LOT 2501832 (n=153)				Seronorm L1 LOT 1706877 (n=190)			
Element	Mean	RSD (%)	Cert. Range	Accuracy (%)	Mean	RSD (%)	Cert. Range	Accuracy (%)
**Li**	5.61	8.9	5.92–8.88	76	5.57	10.9	5.92–8.88	75
**Mg**	44436	5.2	34900–48400	102	43820	7	34900–48400	101
**Ca**	62132	3	54400–81600	91	60542	4.5	54400–81600	89
**V**	0.32	12.4	0.18–0.26	147	0.29	18.5	0.18–0.26	133
**Cr**	6.9	8.4	6.70–9.30	86	7.14	11.9	6.70–9.30	89
**Mn**	**0.34**	**17.6**	**0.24–0.33**	**122**	**0.33**	**18.9**	**0.24–0.33**	**118**
**Fe**	12.9	16.7	7.60–10.6	142	12.9	16.7	7.60–10.6	142
**Co**	0.29	10.2	0.25–0.37	93	0.29	8.9	0.25–0.37	92
**Ni**	1.38	16.6	1.17–1.56	101	1.38	16.6	1.17–1.56	101
**Cu**	94.4	2.1	93.0–119	89	93.9	2.4	93.0–119.0	89
**Zn**	288	12.2	147–195	168	269.1	16.1	147.0–195.0	157
**As**	**33.4**	**2.2**	**29.0–47.0**	**88**	**33.4**	**2.2**	**29.0–47.0**	**88**
**Br**	1458	10.3	1228–1842	95	1473	9.8	1228–1842	96
**Se**	11.1	7.7	10.2–14.7	89	11.2	7.5	10.2–14.7	90
**Sr**	64.3	1.5	54.4–81.6	95	64.2	1.9	54.4–81.6	94
**Mo**	29.5	2.7	24.8–33.0	102	29.5	2.6	24.8–33.0	102
**Cd**	0.081	24.2	0.046–0.068	142	0.082	22.9	0.046–0.068	144
**Sn**	2.38	2.8	2.20–3.10	88	2.37	3.3	2.20–3.10	88
**Sb**	**21.5**	**3.7**	**19.4–30.5**	**86**	**21.4**	**3.8**	**19.4–30.5**	**85**
**Cs**	4.47	1.9	3.52–5.28	102	4.46	2.1	3.52–5.28	101
**Ba**	**65.3**	**2.4**	**54.4–81.6**	**96**	**64.6**	**3.1**	**54.4–81.6**	**95**
**W**	0.057		NC	—	0.058		NC	—
**Tl**	0.16	7.3	0.12–0.17	108	0.15	7.5	0.12–0.17	107
**Pb**	2.21	6.3	1.82–2.42	104	2.13	7.2	1.82–2.42	101

Element (µg/L)	NIST SRM 2668 Level I (n=53)				NIST SRM 2668 Level II (n=55)			
**Element**	**Mean**	**RSD (%)**	**Cert. Range**	**Accuracy (%)**	**Mean**	**RSD (%)**	**Cert. Range**	**Accuracy (%)**
**Li**	13.2		NC	—	12.9		NC	—
**Mg**	42442		NC	—	41771		NC	—
**Ca**	74651		NC	—	72855		NC	—
**V**	0.99	10.4	0.89–1.07	102	46.8	3	43.9–53.1	96
**Cr**	1.21	28.9	0.77–1.39	112	25.3	4.8	25.6–29.8	91
**Mn**	**1.05**	**17.4**	**0.92–1.24**	**97**	**42.6**	**2.3**	**44.2–51.0**	**90**
**Fe**	17.3		NC	—	20.5		NC	—
**Co**	0.68	10.3	0.76–0.87	84	50.2	2.5	50.1–53.5	97
**Ni**	2.62	17.1	1.99–2.63	114	115.6	2.8	110.1–120.5	100
**Cu**	26	12.8	26.1–30.1	93	123.8	4.5	128.7–139.5	92
**Zn**	223.8		NC	—	213.2		NC	—
**As**	**9.74**	**3.4**	**10.3–11.4**	**90**	**199.2**	**2.7**	**208.7–217.5**	**94**
**Br**	2281		NC	—	2286		NC	—
**Se**	23.2		NC	—	21.7		NC	—
**Sr**	79.2		NC	—	77.6		NC	—
**Mo**	50.1	3.8	49.8–53.4	97	1716	2.8	1629–1745	102
**Cd**	1.07	6.4	1.00–1.11	101	16.2	2.9	16.1–16.6	99
**Sn**	1.35	10	1.55–1.83	80	149.2	4	162.0–180.0	87
**Sb**	**0.19**	**17**	**0.21–0.27**	**80**	**19.2**	**7.5**	**21.4–23.4**	**86**
**Cs**	4.84	3.6	4.60–5.20	99	219.7	2.4	209.0–233.0	99
**Ba**	**1.93**	**16.1**	**1.82–2.10**	**98**	**241.8**	**5**	**251.4–257.8**	**95**
**W**	1.13	12.2	1.17–1.33	90	56.6	4	61.5–63.5	91
**Tl**	0.65	4.3	0.69–0.75	90	102.2	2.7	112.4–118.0	89
**Pb**	1.31	18.9	1.17–1.29	107	123.7	2.4	134.3–141.5	90

Element (µg/L)	ClinChek L1 (RECIPE) (n=102)				QMEQAS QM-U-Q2313 (n=144)			
Element	Mean	RSD (%)	Cert. Range	Accuracy (%)	Mean	RSD (%)	Cert. Range	Accuracy (%)
**Li**	4.55		NC	—	100.5	6.5	86.4–129.6	93
**Mg**	18499	4.9	14900–22300	100	55251		NC	—
**Ca**	15175	7.3	13840–20760	88	81389		NC	—
**V**	18.1	8.3	16.8–25.2	88	1.37	3.5	1.22–1.82	91
**Cr**	3.41	12.3	3.26–4.90	82	1.38	19.5	1.10–1.66	100
**Mn**	**3.4**	**6**	**3.27–4.90**	**86**	**2.25**	**5.2**	**1.99–2.99**	**91**
**Fe**	33.8	12.5	32.5–48.8	85	28.2		NC	—
**Co**	1.81	9	1.64–2.46	92	11.3	1.9	9.36–14.0	97
**Ni**	3.47	17.8	2.60–3.90	103	9.11	8	6.96–10.4	105
**Cu**	68.2	31.2	46.6–69.9	129	157.9	4.1	131.2–196.8	96
**Zn**	178.4	10.5	156.0–234.0	100	401.8	2.5	318.4–477.6	101
**As**	**15.2**	**8.3**	**13.6–20.3**	**91**	**43**	**1.8**	**37.9–56.9**	**91**
**Br**	329.8		NC	—	1949		NC	—
**Se**	19.3	10	21.8–36.3	75	89.3	3.2	85.6–128.4	83
**Sr**	27.1		NC	—	106.9	1.2	91.2–136.8	94
**Mo**	19.4	9.6	16.2–24.3	100	101.5	2.4	79.8–119.6	102
**Cd**	2.13	10.7	2.05–3.06	97	8.68	3	6.84–10.3	102
**Sn**	4.29	8.1	4.13–6.19	84	6.57	7	4.88–7.32	108
**Sb**	**5.09**	**8.7**	**4.88–7.31**	**95**	**2.83**	**3.9**	**2.67–3.59**	**90**
**Cs**	0.81		NC	—	13.3	1.7	10.6–15.8	101
**Ba**	**9.76**	**7.5**	**8.77–13.2**	**90**	**8.08**	**2.9**	**6.63–9.95**	**97**
**W**	0.076		NC	—	0.19		NC	—
**Tl**	6.6	8.3	5.90–8.85	93	8.97	2.8	7.46–11.2	96
**Pb**	21.7	7.1	21.1–31.6	80	103.9	3.9	86.4–129.6	96

Element (µg/L)	QMEQAS QM-U-Q2315 (n=112)			
**Element**	**Mean**	**RSD (%)**	**Cert. Range**	**Accuracy (%)**
**Li**	155.6	8	130.4–195.6	96
**Mg**	66079		NC	—
**Ca**	80259		NC	—
**V**	5.94	2.1	5.12–7.68	93
**Cr**	4.29	9.6	3.94–5.92	87
**Mn**	**7.7**	**2.4**	**6.94–10.4**	**89**
**Fe**	14.9		NC	—
**Co**	11.1	2	9.04–13.6	99
**Ni**	5.75	21.1	4.26–6.40	108
**Cu**	55.2	3.7	46.2–69.4	96
**Zn**	577	1.8	460.8–691.2	100
**As**	**30.5**	**2**	**26.7–40.1**	**91**
**Br**	2193		NC	—
**Se**	25.6	5.9	30.2–45.2	68
**Sr**	98.8	1.1	84.0–126.0	94
**Mo**	153.7	2.6	120.0–180.0	102
**Cd**	4.94	3.1	4.05–6.07	98
**Sn**	15.1	4.6	14.6–21.8	83
**Sb**	**1.54**	**4.2**	**1.47–2.03**	**88**
**Cs**	6.02	1.9	4.72–7.08	102
**Ba**	**10.4**	**2.8**	**8.64–13.0**	**97**
**W**	0.052		NC	—
**Tl**	3.45	3.3	2.85–4.27	97
**Pb**	346.8	2.5	289.6–434.4	96

Table S10. Performance characteristics for certified urine reference materials used for quality assurance and quality control of urinary metal measurements. This table summarizes the analytical performance of certified reference materials (CRMs) and external quality assessment samples used to evaluate the accuracy and precision of urinary metal measurements throughout the study. Reference materials included Seronorm™ Trace Elements Urine (Levels 1), NIST Standard Reference Material (SRM) 2668 Toxic Elements in Frozen Human Urine (Levels I and II), ClinChek® Urine Control (Level 1), and samples from the Quebec Multielement External Quality Assessment Scheme (QMEQAS). For each analyte, the mean measured concentration, relative standard deviation (RSD), certified or assigned concentration range, and analytical accuracy are reported. Accuracy was calculated as the percentage agreement between the measured concentration and the certified or assigned target value. These results demonstrate the reliability, reproducibility, and analytical validity of the inductively coupled plasma mass spectrometry (ICP-MS) method used for urinary metal quantification. Abbreviations: RSD, relative standard deviation; CRM, certified reference material; NC, no certified concentration available; NIST, National Institute of Standards and Technology; QMEQAS, Quebec Multielement External Quality Assessment Scheme.

Metal	Median MauiWES urine (µg/L)	2 mL Corning™ Polypropylene Cryogenic Vials n = 12			100 mL Urine Container, Needle Yellow Cap (Avantor/VWR) n = 9		
		Median (µg/L)	SD (µg/L)	% of MauiWES urine median	Median (µg/L)	SD (µg/L)	% of MauiWES urine median
**Li**	19.4	-0.007	0.012	*<0.01%*	0.001	0.013	*<0.01%*
**Mg**	52300	6.10	2.1	0.01%	20.3	18.0	0.04%
**Ca**	89000	270	70	0.30%	980	890	**1.10%**
**V**	0.22	0.012	0.004	**5.50%**	0.005	0.010	**2.05%**
**Cr**	1.43	-0.006	0.019	*<0.01%*	0.07	0.143	**4.95%**
**Mn**	0.46	0.017	0.012	**3.70%**	0.040	0.031	**8.90%**
**Fe**	14.5	-0.014	0.130	*<0.01%*	0.317	0.370	**2.19%**
**Co**	0.35	0.001	0.003	0.23%	0.000	0.003	*<0.01%*
**Ni**	2.23	0.027	0.026	**1.20%**	0.046	0.045	**2.04%**
**Cu**	10.3	0.013	0.066	0.12%	0.039	0.182	0.38%
**Zn**	609	0.390	1.30	0.06%	9.85	6.75	**1.62%**
**As**	18.1	-0.001	0.003	*<0.01%*	-0.001	0.003	*<0.01%*
**Br**	2680	1.100	2.900	0.04%	1.69	2.18	0.06%
**Se**	45.2	-0.019	0.029	*<0.01%*	-0.008	0.160	*<0.01%*
**Sr**	125	0.250	0.120	0.20%	0.510	0.480	0.41%
**Mo**	46.0	-0.027	0.013	*<0.01%*	-0.060	0.041	*<0.01%*
**Pd**	0.25	-0.029	0.007	*<0.01%*	-0.052	0.056	*<0.01%*
**Cd**	0.23	0.001	0.002	0.24%	0.001	0.002	0.33%
**Sn**	0.59	-0.007	0.003	*<0.01%*	0.006	0.011	**1.04%**
**Sb**	1.97	-0.001	0.001	*<0.01%*	0.001	0.004	0.05%
**Cs**	5.87	0.000	0.000	<0.01%	0.001	0.001	<0.01%
**Ba**	2.56	0.010	0.021	0.37%	0.067	0.091	**2.62%**
**W**	0.18	0.001	0.004	0.43%	0.0003	0.002	0.17%
**Tl**	0.18	-0.001	0.000	*<0.01%*	0.001	0.003	0.47%
**Pb**	0.39	0.001	0.11	0.27%	-0.005	0.026	*<0.01%*
**Bi**	0.005	-0.006	0.001	*<0.01%*	-0.005	0.003	*<0.01%*
**U**	0.008	-0.002	0.001	*<0.01%*	-0.001	0.002	*<0.01%*

Table S11. Background metal concentrations in urine collection vials and estimated contribution to median MauiWES urine concentrations (N = 1,647). Values shown as *<0.01%* indicate that the background-subtracted leachate concentration was close to zero or negative because the ICP-MS signal was indistinguishable from the procedural blank within analytical uncertainty. These values indicate no measurable contribution from the vial or collection container to urinary metal concentrations.

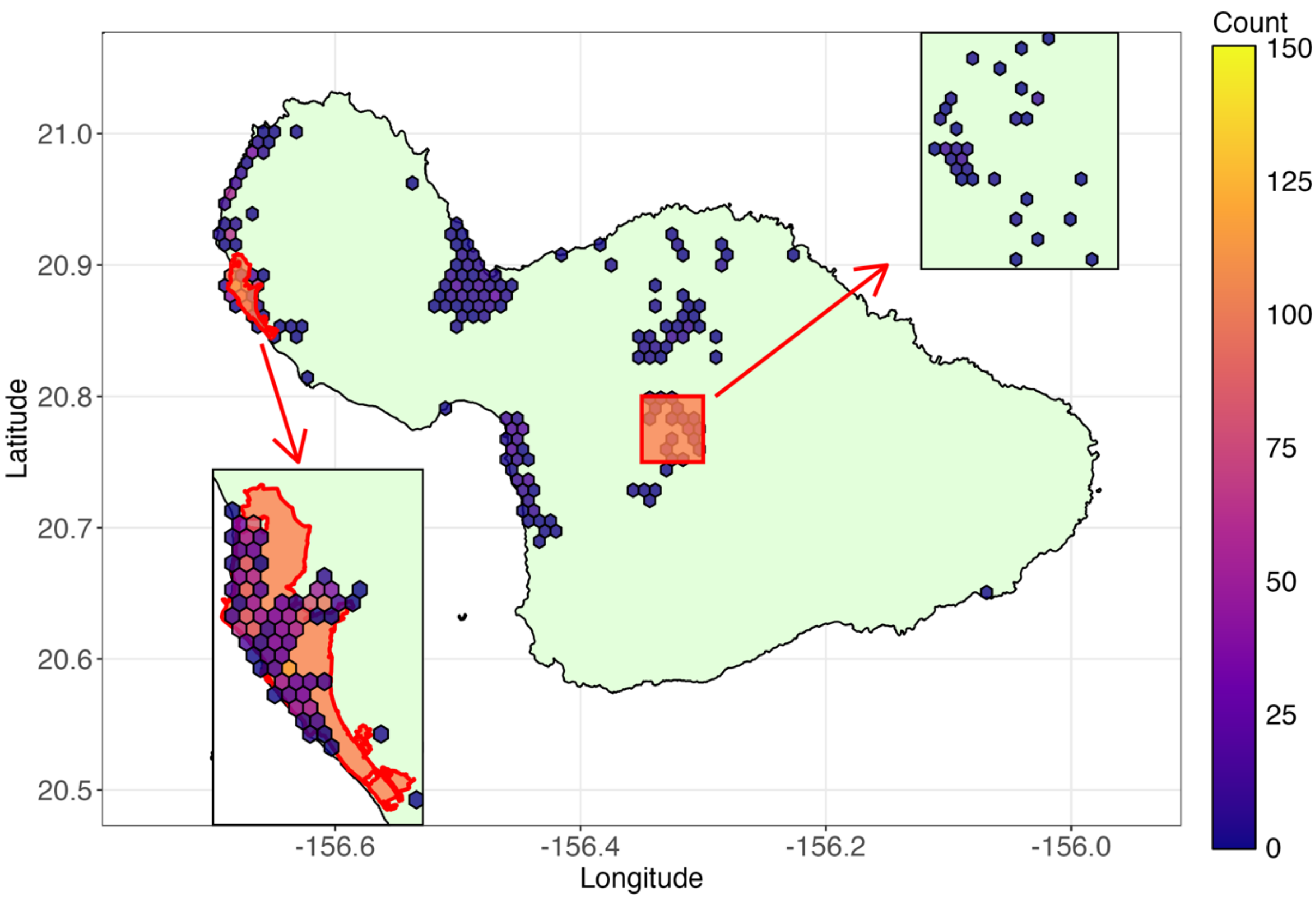

Figure S1. **Geographic distribution of participants enrolled in the Maui Wildfire Exposure Study (MauiWES).** The orange-shaded areas indicate the Lahaina and Kula wildfire burn perimeters. Hexagonal bins depict the spatial distribution of participant residential during the first year of study enrollment, with lighter shading corresponding to a greater number of participants within each hexagonal bin. The figure illustrates the geographic coverage of the MauiWES cohort across Maui.

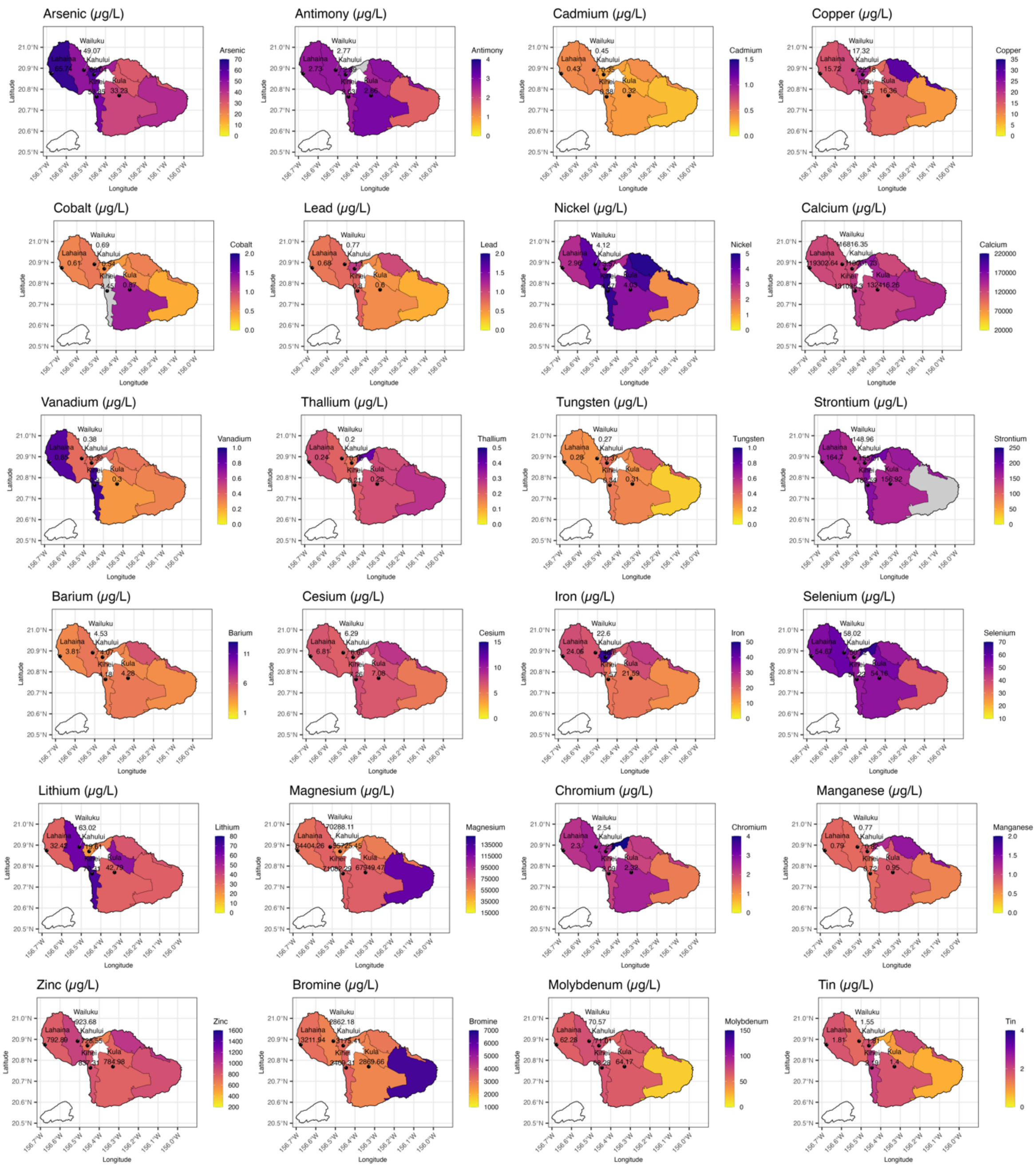

Figure S2. Geographic distribution of urinary metal concentrations among MauiWES participants. Maps display mean urinary concentrations (µg/L), normalized for urine specific gravity, for 24 metals measured in MauiWES: arsenic, antimony, cadmium, copper, cobalt, lead, nickel, calcium, vanadium, thallium, tungsten, strontium, barium, cesium, iron, selenium, lithium, magnesium, chromium, manganese, zinc, bromine, molybdenum, and tin. Gray color indicates missing information in the region. Based on participants with available urinary metal data (analytic N = 1,618), regional values were shown for five primary population areas of Maui: Lahaina (N = 1,041), Kula (N = 139), Kahului (N = 117), Kihei (N = 182), and Wailuku (N = 139). Color gradients represent region-level mean concentrations, with darker intensities indicating higher levels. These maps illustrate substantial geographic heterogeneity in metal exposure profiles across the island.

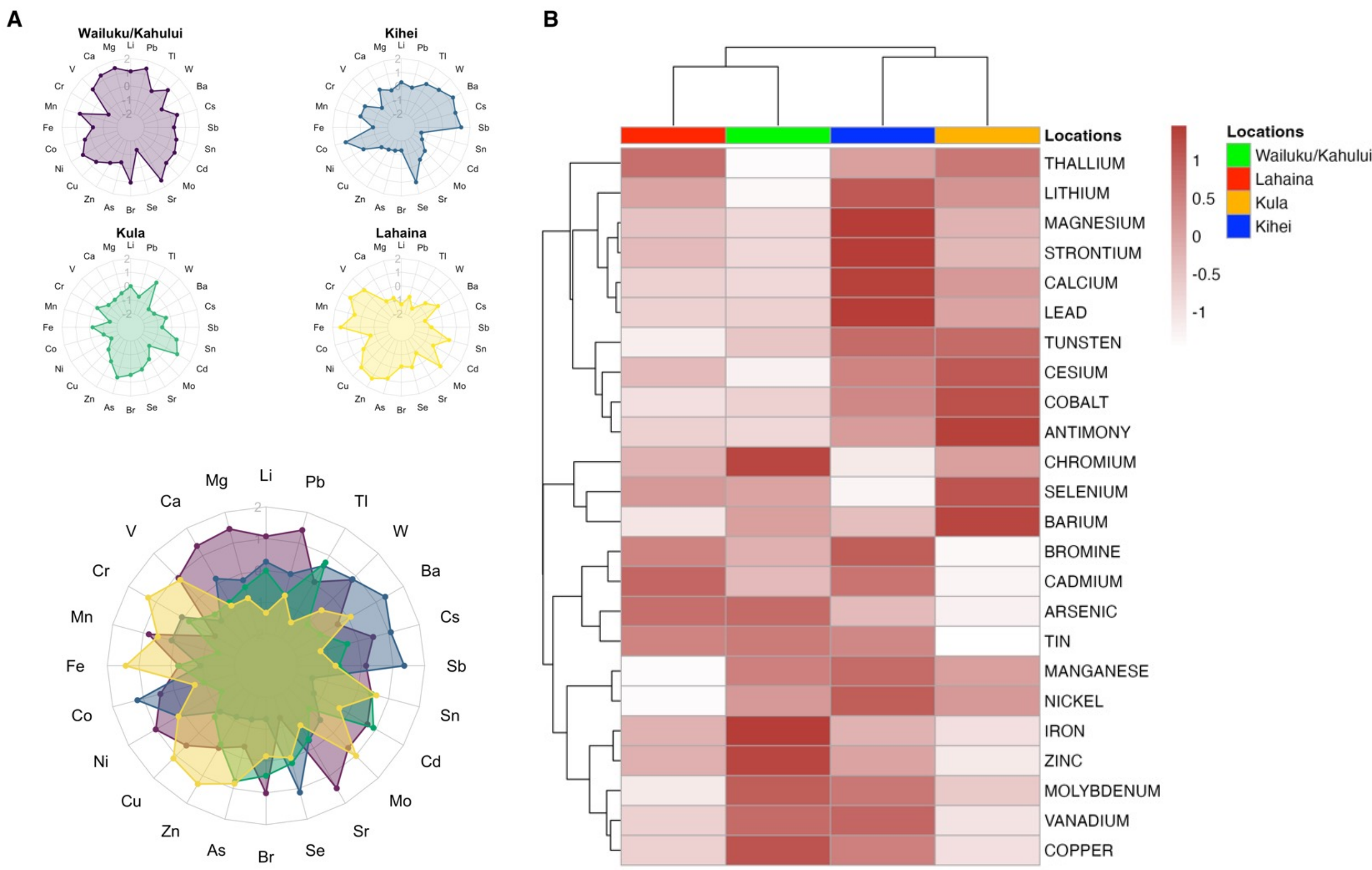

Figure S3. Regional patterns of urinary metal concentrations across Maui. (**A**) Radar plots depicting median urinary concentrations of 24 metals across four major Maui regions. (**B**) Clustered heatmap of standardized median metal concentrations grouped by similarity in exposure profiles. For visualization, urinary metal concentrations corrected for urine specific gravity were natural log-transformed and z-score standardized across the full cohort. Kahului/Wailuku and Kihei exhibited similar exposure patterns, whereas Lahaina and Kula formed distinct clusters, indicating regional heterogeneity in metal burdens consistent with differential wildfire-related exposures. Regional sample sizes were Lahaina (N = 918), Kahului/Wailuku (N = 184), Kihei (N = 164), and Kula (N = 134).

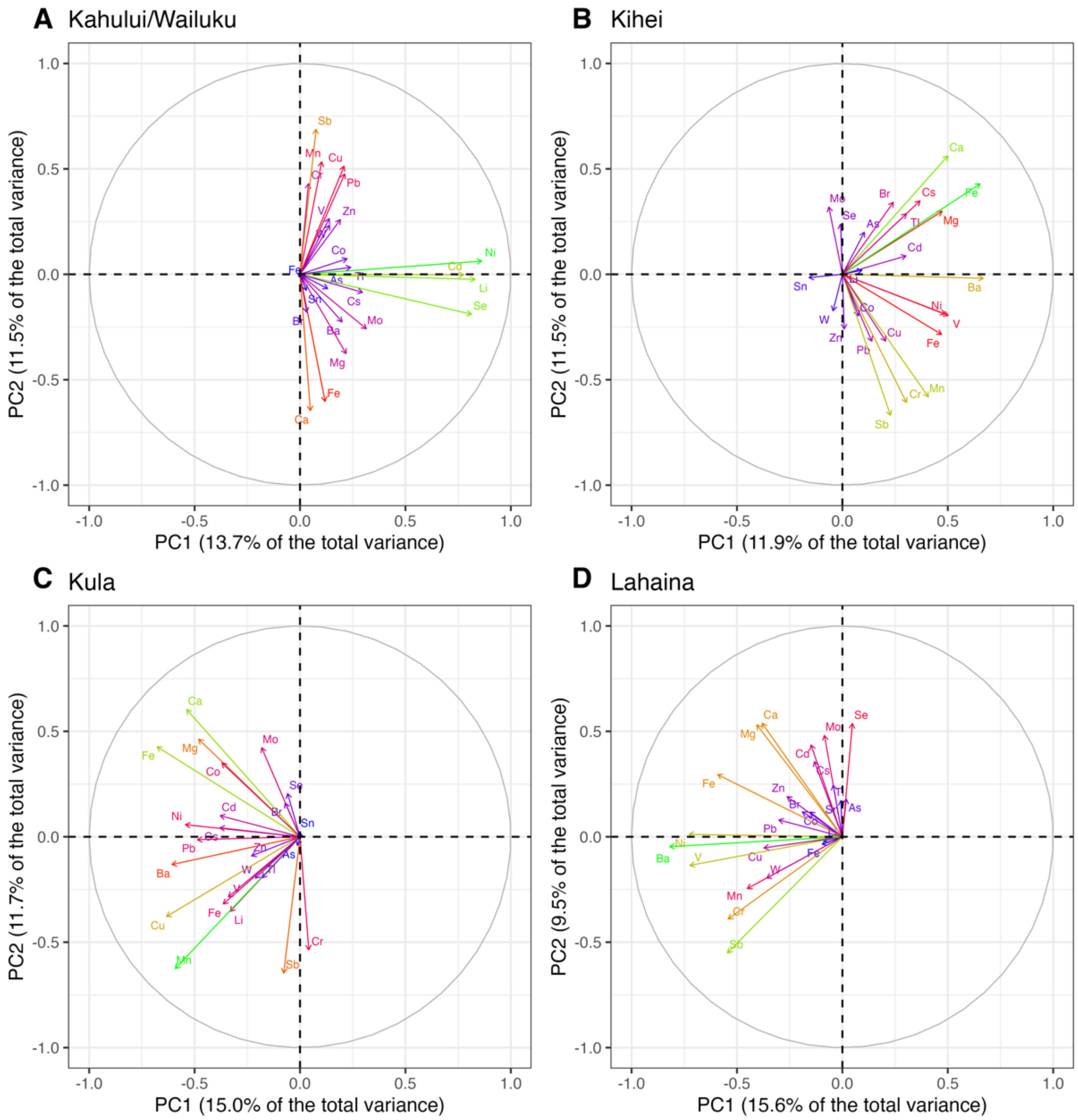

Figure S4. Principal component analysis (PCA) biplots of regional urinary metal profiles across Maui. Panels depict the direction and relative contribution of urinary metals to the first two principal components (PC1 and PC2) within (**A**) Kahului/Wailuku, (**B**) Kihei, (**C**) Kula, and (**D**) Lahaina. Urinary metal concentrations corrected for urine specific gravity were natural log-transformed and z-score standardized across the full cohort prior to region-specific PCA. Each panel displays loading vectors for all measured metals on PC1 and PC2. Kahului/Wailuku and Kihei exhibited broadly similar loading patterns, whereas Lahaina and Kula demonstrated distinct configurations, consistent with region-specific differences in metal exposure profiles. Regional sample sizes were Lahaina (N = 918), Kahului/Wailuku (N = 184), Kihei (N = 164), and Kula (N = 134).

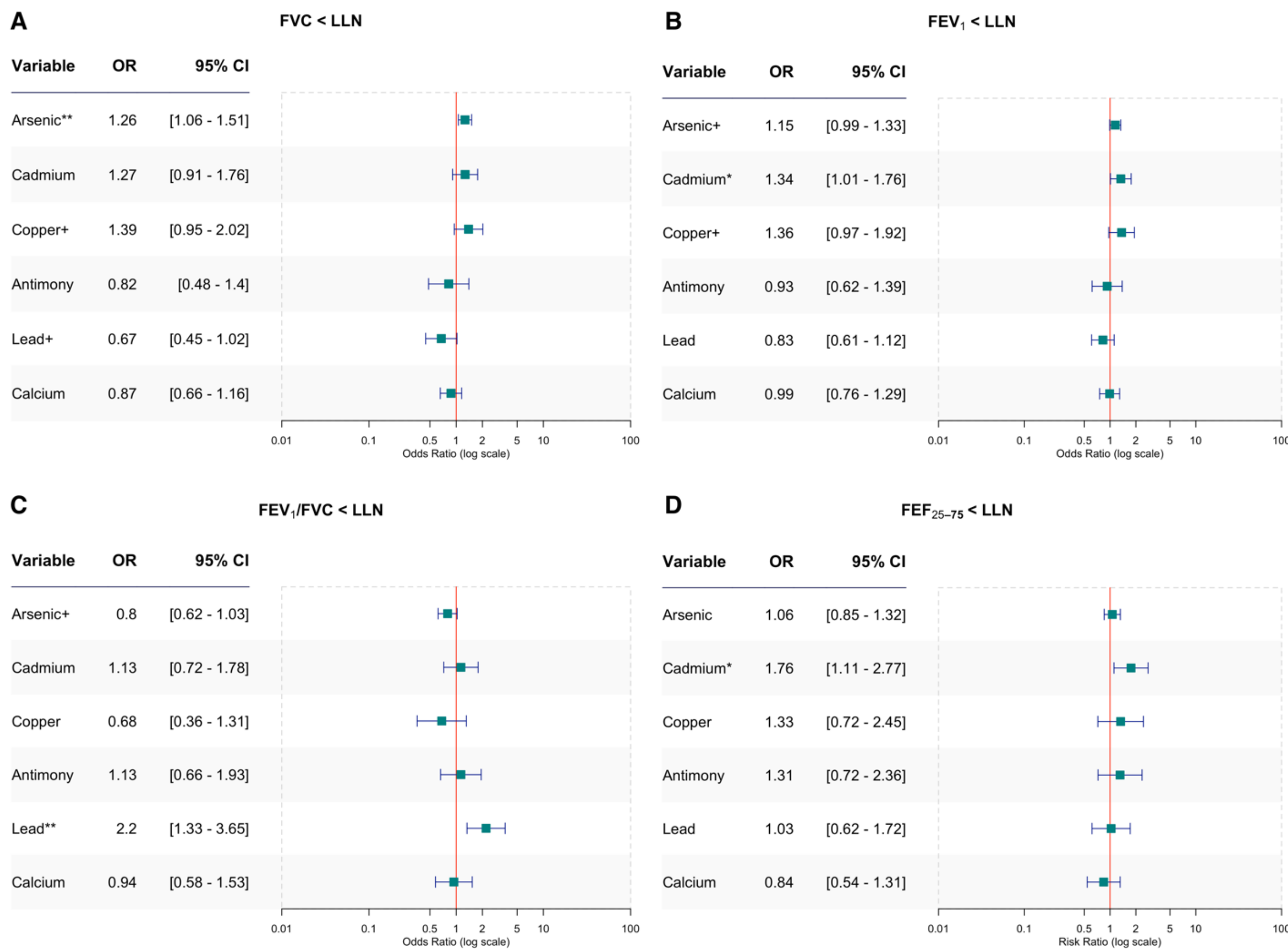

Figure S5. Associations between individual urinary metals and abnormal pulmonary function in adjusted generalized linear models. Each panel displays odds ratios (ORs) and 95% confidence intervals (CIs) for the relationship between a single metal and the likelihood of spirometry values falling below the LLN for FVC (**A**), FEV_1 (**B**), FEV_1/FVC (**C**), and $FEF_{25\text{–}75}$ (**D**). Models were adjusted for age, sex, smoking status, body mass index, and residential region (Lahaina, Kihei, Kahului/Wailuku), with Kula used as the reference category. Analyses included spirometry tests meeting ATS/ERS quality grades A–C. Sample sizes varied by outcome: FEV_1 < LLN (N = 790), FVC < LLN (N = 737), and FEV_1/FVC < LLN and $FEF_{25\text{–}75}$ < LLN (N = 624). Statistical significance is indicated as follows: + $p < 0.10$; * $p < 0.05$; ** $p < 0.01$; *** $p < 0.001$.

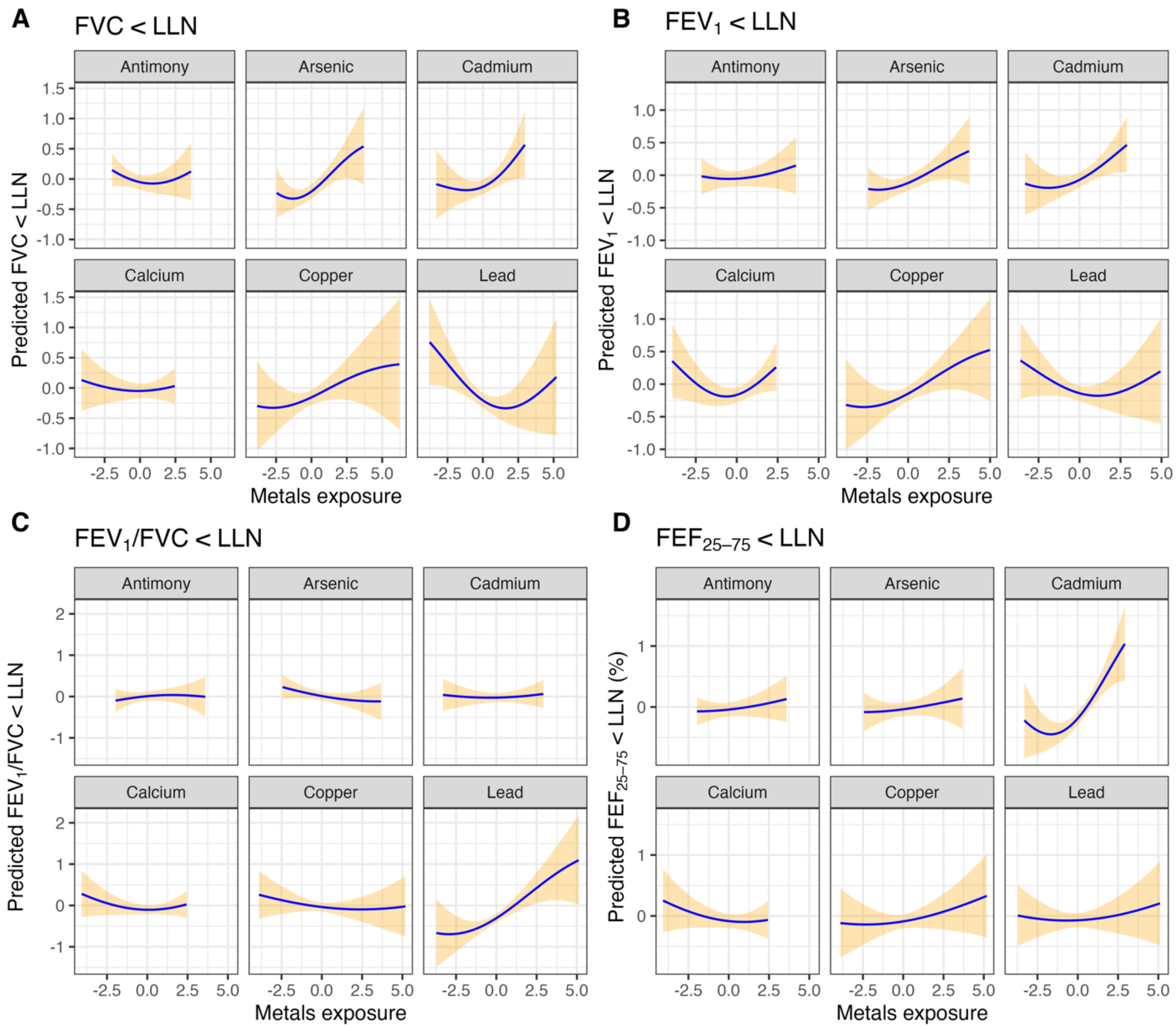

Figure S6. Univariate exposure–response functions for individual urinary metals estimated using BKMR. Each curve depicts the predicted log-odds of abnormal pulmonary outcomes as a function of standardized metal exposure, holding all other metals at their median values. Panels show effects on (**A**) FVC < LLN, (**B**) FEV_1 < LLN, (**C**) FEV_1/FVC < LLN, and (**D**) $FEF_{25–75}$ < LLN. Shaded areas represent 95% credible intervals. The models were adjusted for age, sex, smoking status, and body mass index, and included spirometry meeting ATS/ERS quality grades A–C. Sample sizes varied by outcome: FEV_1 < LLN (N = 790), FVC < LLN (N = 737), and FEV_1/FVC < LLN and $FEF_{25–75}$ < LLN (N = 624).

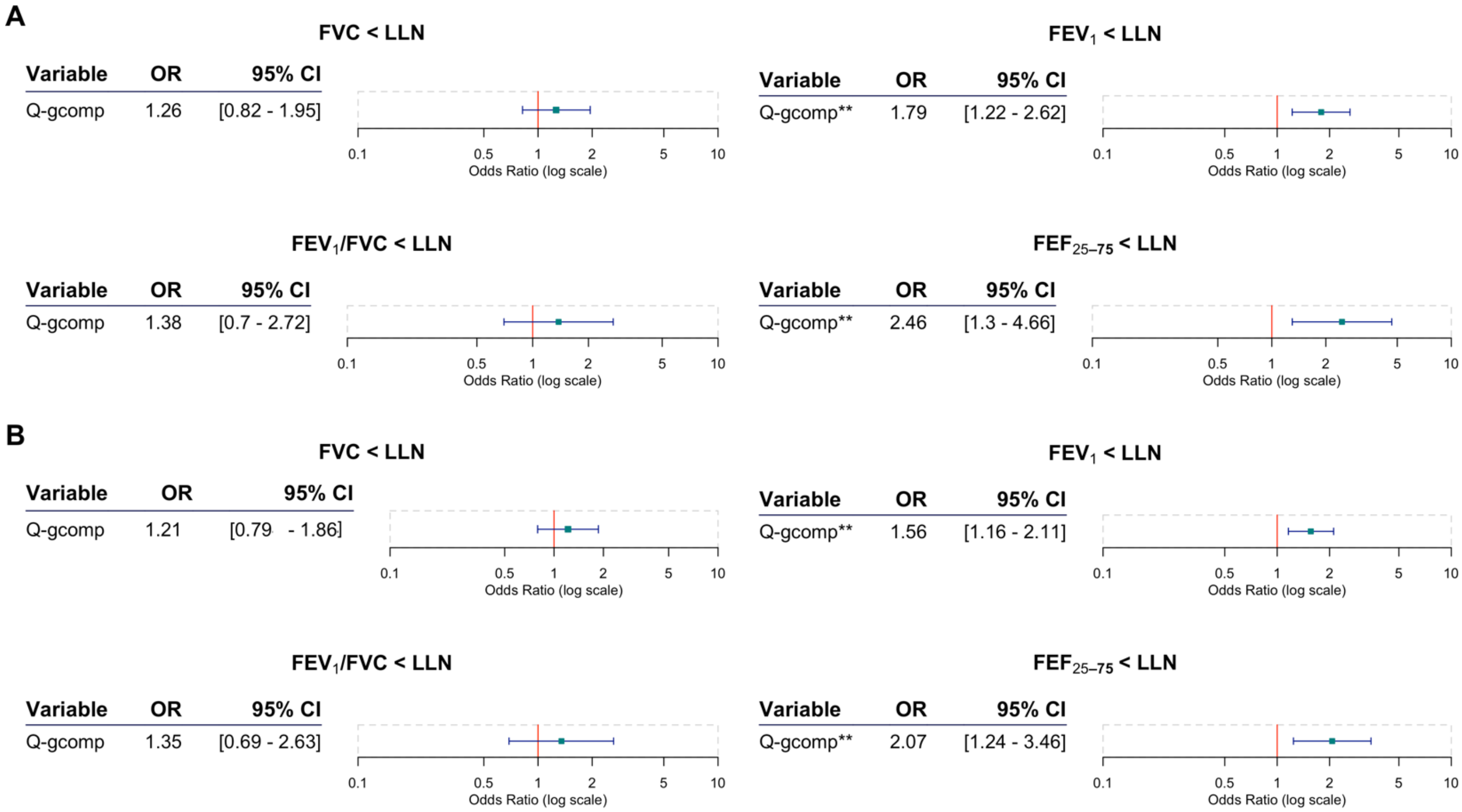

Figure S7. Associations between metal mixtures and abnormal pulmonary function estimated using quantile g-computation (Q-gComp) models with and without bootstrapping. (**A**) Q-gComp model estimates without bootstrapping. (**B**) Q-gComp model estimates with 500 bootstrap iterations. Models were adjusted for age, sex, smoking status, and BMI and restricted to spirometry tests meeting ATS/ERS quality grades A–C in the full MauiWES cohort. Outcomes were modeled as binary indicators of FEV_1 < LLN, FVC < LLN, FEV_1/FVC < LLN, and $FEF_{25–75}$ < LLN. Sample sizes varied by outcome: FEV_1 < LLN (N = 790), FVC < LLN (N = 737), and FEV_1/FVC < LLN and $FEF_{25–75}$ < LLN (N = 624). Results are presented as odds ratios (ORs) with 95% confidence intervals (CIs). Statistical significance is indicated as follows: +*p* < 0.10; **p* < 0.05; ***p* < 0.01; ****p* < 0.001.

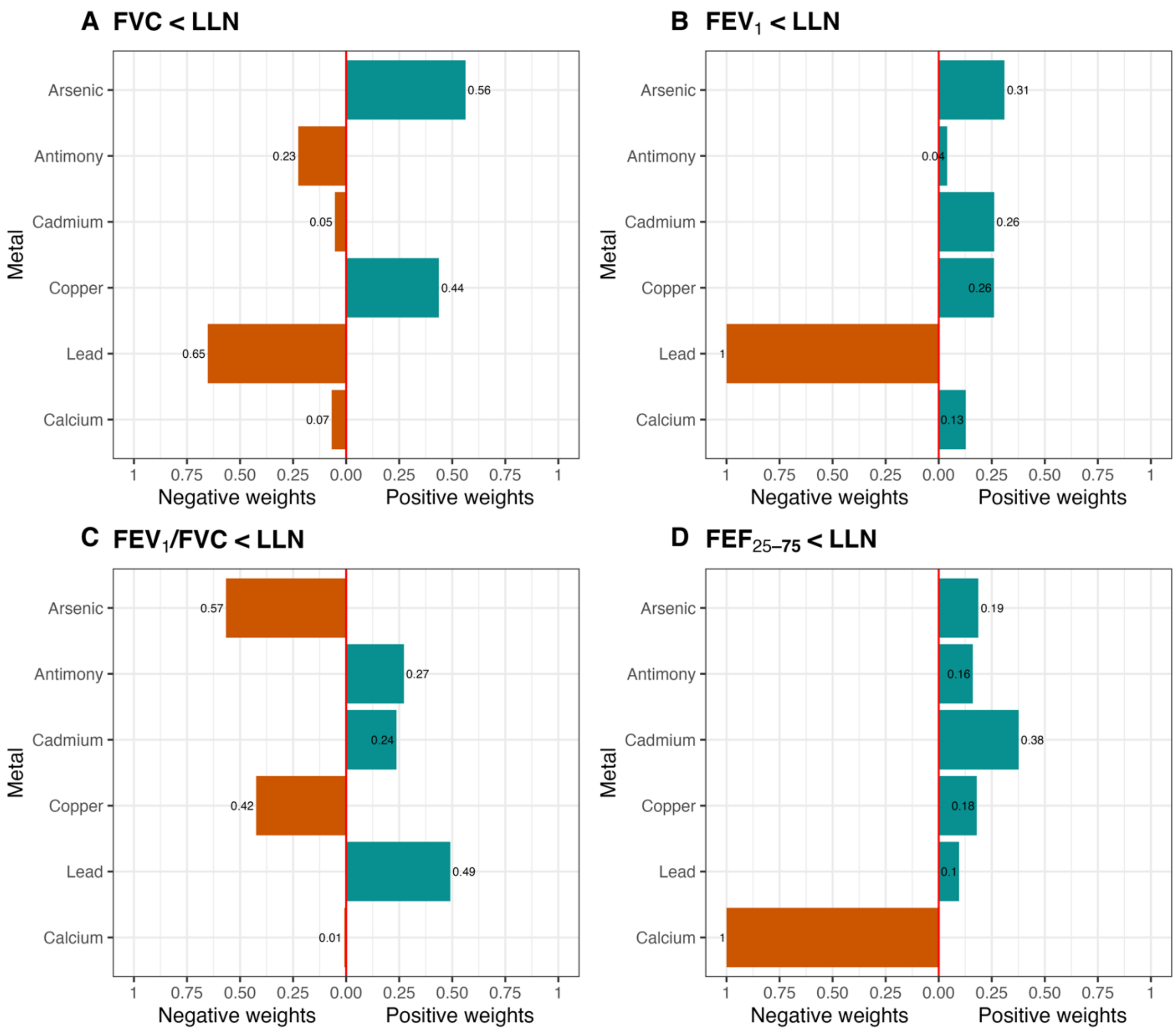

Figure S8. Q-gComp model weights for individual metals contributing to mixture effects across pulmonary outcomes. Panels depict the relative contribution and directionality of individual metals associated with (**A**) FVC < LLN, (**B**) FEV_1 < LLN, (**C**) FEV_1/FVC < LLN, and (**D**) $FEF_{25–75}$ < LLN. Bars represent estimated weights assigned to each metal in the positive (right) and negative (left) directions of the overall mixture effect in non-bootstrapped Q-gComp models. Models were adjusted for age, sex, smoking status, and BMI and restricted to spirometry tests meeting ATS/ERS quality grades A–C. Sample sizes varied by outcome: FEV_1 < LLN (N = 790), FVC < LLN (N = 737), and FEV_1/FVC < LLN and $FEF_{25–75}$ < LLN (N = 624).

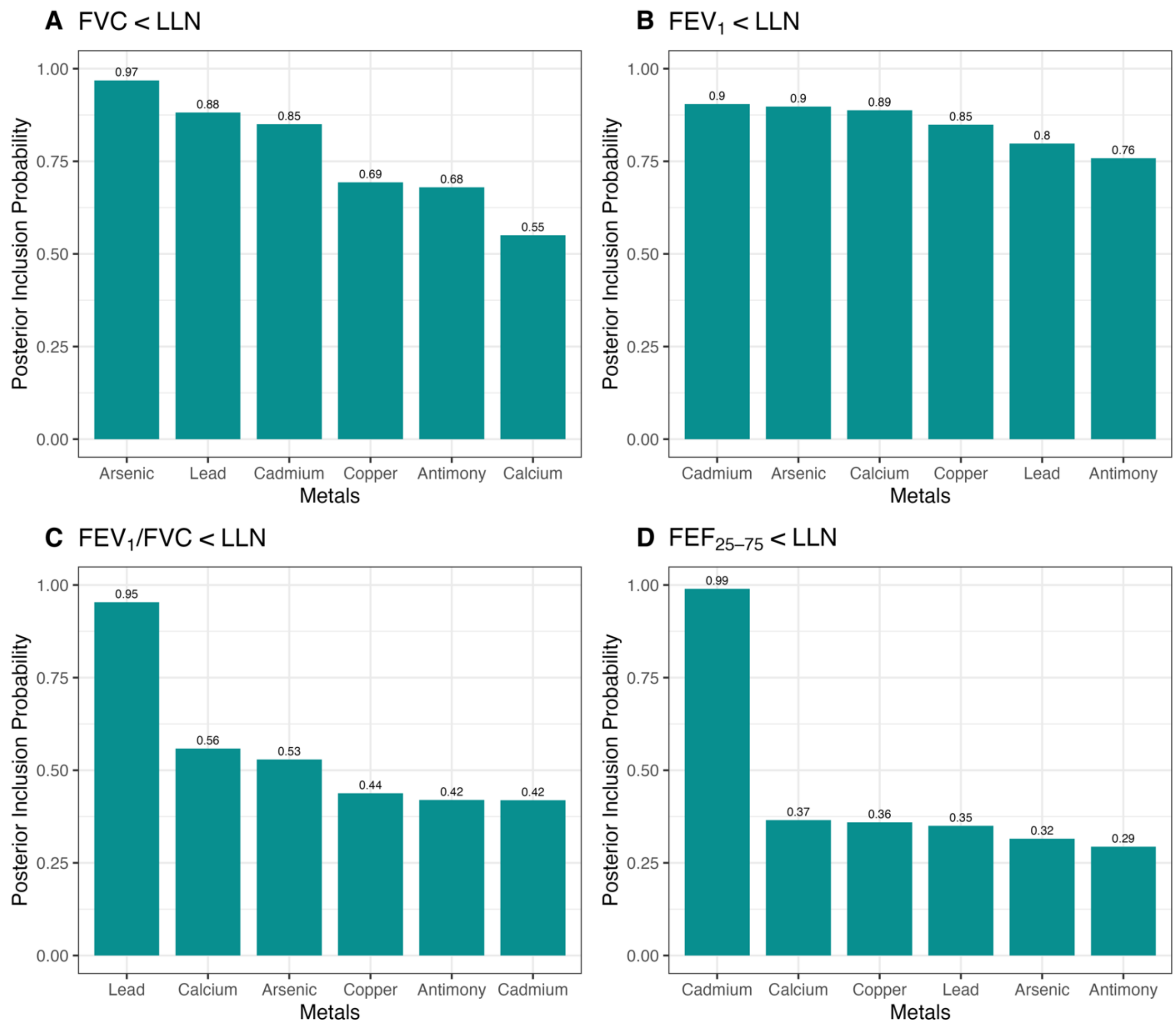

Figure S9. Posterior inclusion probabilities (PIPs) for individual metals in Bayesian kernel machine regression (BKMR) models evaluating associations between metal mixtures and impaired lung function. Panels show PIPs for metals associated with (**A**) FVC < LLN, (**B**) FEV_1 < LLN, (**C**) FEV_1/FVC < LLN, and (**D**) $FEF_{25–75}$ < LLN. Higher PIPs indicate greater relative importance of a metal in explaining variation in each pulmonary outcome. Models were adjusted for age, sex, smoking status, and BMI and restricted to spirometry tests meeting ATS/ERS quality grades A–C. Sample sizes varied by outcome: FEV_1 < LLN (N = 790), FVC < LLN (N = 737), and FEV_1/FVC < LLN and $FEF_{25–75}$ < LLN (N = 624).

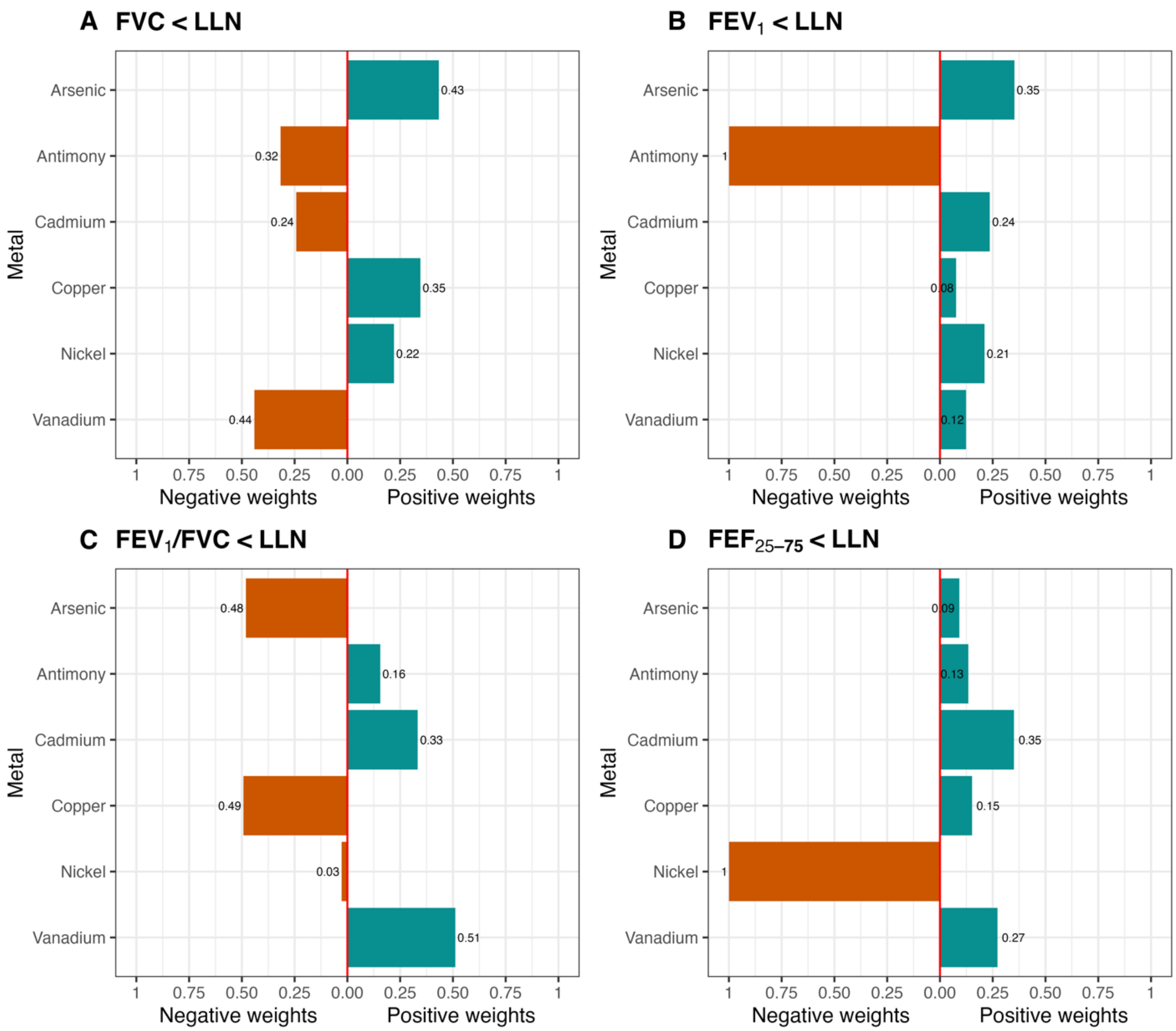

Figure S10. Positive and negative weight contributions of individual metals in Q-gComp models for the Lahaina and Kula regions. Panels depict the direction and relative magnitude of metal-specific contributions associated with (**A**) FVC < LLN, (**B**) FEV_1 < LLN, (**C**) FEV_1/FVC < LLN, and (**D**) $FEF_{25–75}$ < LLN. Bars represent the estimated positive and negative contributions of each metal to the overall mixture effect for each pulmonary outcome. Models were adjusted for age, sex, smoking status, and BMI and restricted to spirometry tests meeting ATS/ERS quality grades A–C. Sample sizes varied by outcome: FEV_1 < LLN (N = 587), FVC < LLN (N = 537), and FEV_1/FVC < LLN and $FEF_{25–75}$ < LLN (N = 460).

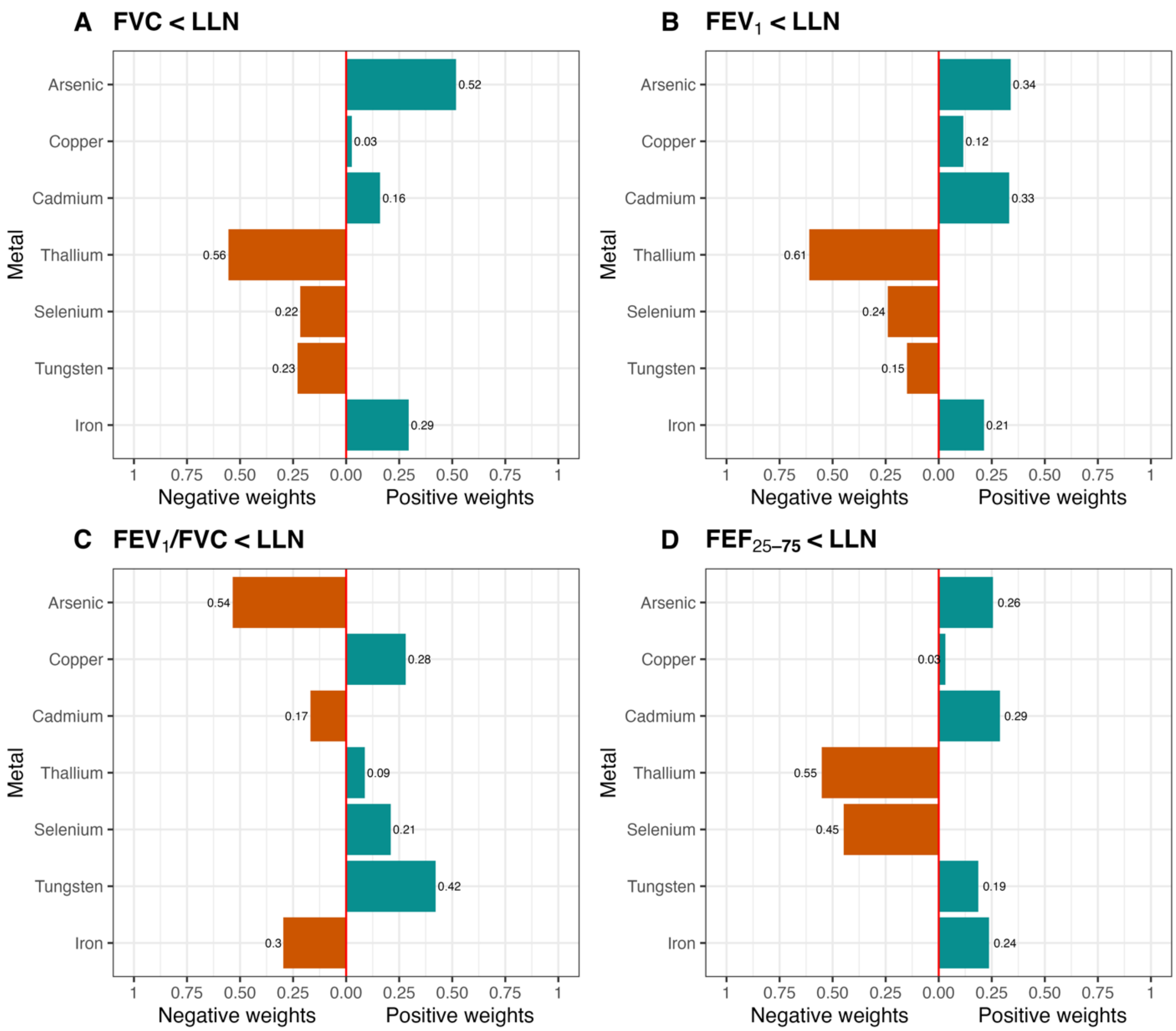

Figure S11. Positive and negative weight contributions of individual metals in Q-gComp models for the Wailuku, Kahului, and Kihei regions. Panels depict the direction and relative magnitude of metal-specific contributions associated with (**A**) FVC < LLN, (**B**) FEV_1 < LLN, (**C**) FEV_1/FVC < LLN, and (**D**) $FEF_{25–75}$ < LLN. Bars represent the estimated positive and negative contributions of each metal to the overall mixture effect for each pulmonary outcome. Models were adjusted for age, sex, smoking status, and BMI and restricted to spirometry tests meeting ATS/ERS quality grades A–C. Sample sizes varied by outcome: FEV_1 < LLN (N = 203), FVC < LLN (N = 200), and FEV_1/FVC < LLN and $FEF_{25–75}$ < LLN (N = 164).

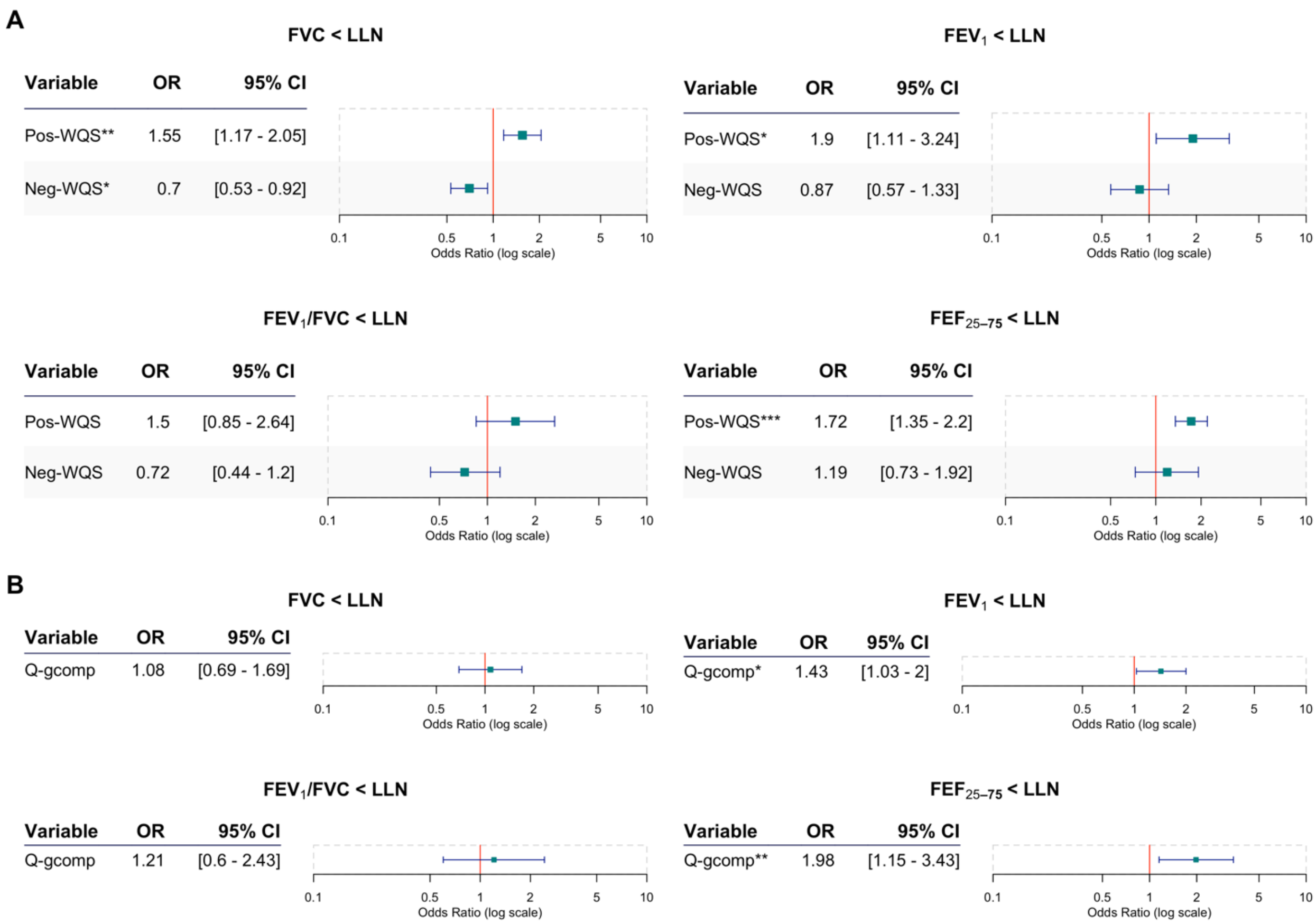

Figure S12. Associations between metal mixtures and impaired lung function among non-smokers. (**A**) Estimates from dual-index weighted quantile sum (WQS) regression models. (**B**) Estimates from quantile g-computation (Q-gComp) models with 500 bootstrap iterations. Models were adjusted for age, sex, and BMI and restricted to non-smokers with spirometry tests meeting ATS/ERS quality grades A–C in the MauiWES cohort. Outcomes were modeled as binary indicators of FEV_1 < LLN, FVC < LLN, FEV_1/FVC < LLN, and $FEF_{25–75}$ < LLN. Sample sizes varied by outcome: FEV_1 < LLN (N = 659), FVC < LLN (N = 612), and FEV_1/FVC < LLN and $FEF_{25–75}$ < LLN (N = 526). Results are presented as odds ratios (ORs) with 95% confidence intervals (CIs). Statistical significance is indicated as follows: +$p < 0.10$; *$p < 0.05$; **$p < 0.01$; ***$p < 0.001$.

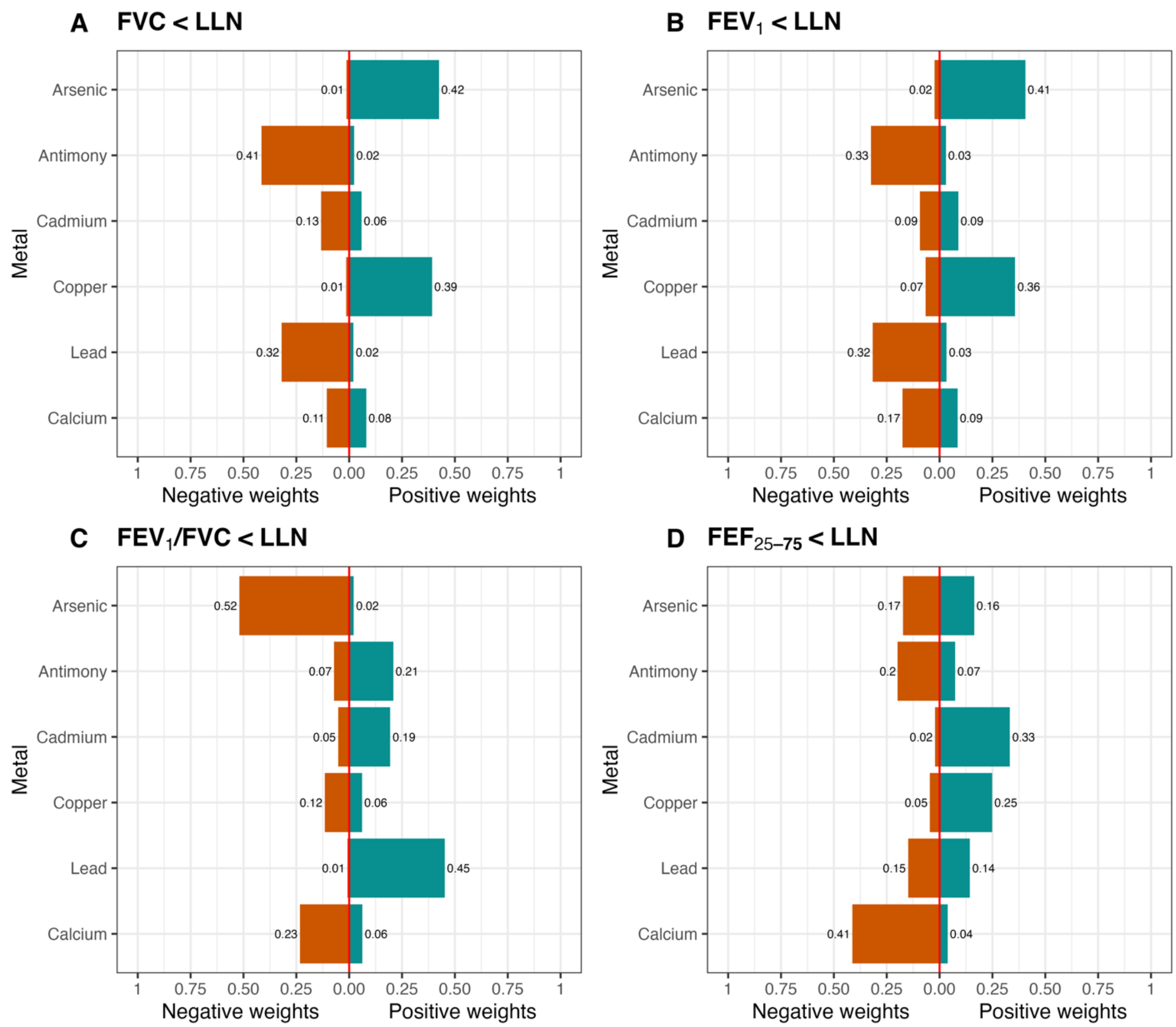

Figure S13. Positive and negative weight contributions of individual metals in dual-index weighted quantile sum (WQS) models among non-smokers. Panels depict the direction and relative magnitude of metal-specific contributions associated with (**A**) FVC < LLN, (**B**) FEV_1 < LLN, (**C**) FEV_1/FVC < LLN, and (**D**) $FEF_{25–75}$ < LLN. Bars represent the estimated positive and negative contributions of each metal to the overall mixture effect for each pulmonary outcome. Models were adjusted for age, sex, and BMI and restricted to non-smokers with spirometry tests meeting ATS/ERS quality grades A–C in the MauiWES cohort. Sample sizes varied by outcome: FEV_1 < LLN (N = 659), FVC < LLN (N = 612), and FEV_1/FVC < LLN and $FEF_{25–75}$ < LLN (N = 526).

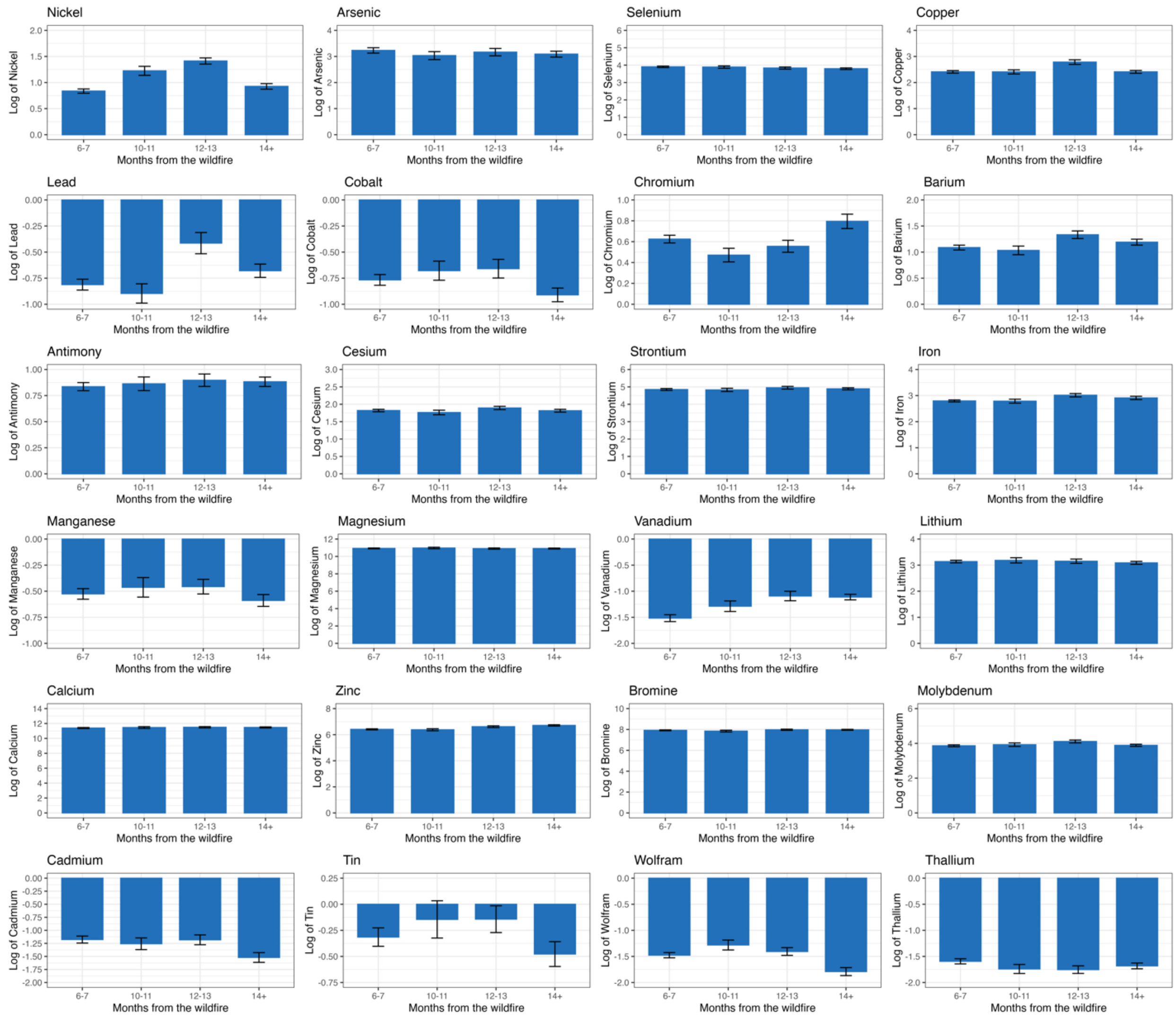

Figure S14. **Urinary metal concentrations by time interval between the Maui wildfire event and biospecimen collection.** Urinary metal concentrations (log-transformed) are shown according to the interval between the August 2023 Maui wildfire and biospecimen collection. Bars represent mean concentrations within each sampling interval, and error bars indicate 95% CI. Concentrations varied across post-wildfire sampling intervals for several metals. Metals such as arsenic, copper, antimony, and lead exhibited relatively similar concentrations across collection periods, whereas cadmium, tin, and tungsten (wolfram) tended to be lower among participants sampled at later intervals following the wildfire event. Because participants were sampled at a single time point rather than followed longitudinally, these results reflect cross-sectional differences among participants enrolled at different times after the wildfire and should not be interpreted as within-person changes over time.

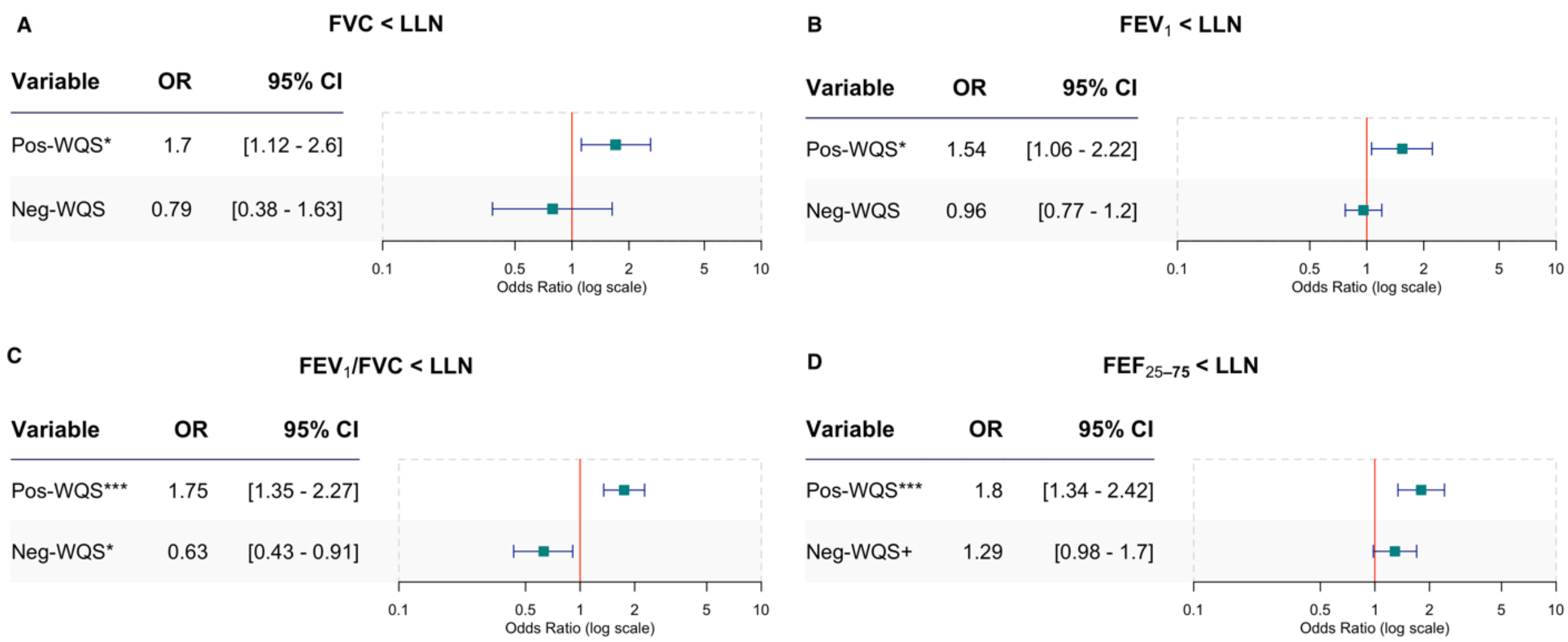

Figure S15. Sensitivity analysis of the associations between metal mixture exposure and abnormal pulmonary function estimated using a dual-index WQS model controlling for time since the fire. Panels depict associations with (**A**) FVC < LLN, (**B**) FEV_1 < LLN, (**C**) FEV_1/FVC < LLN, and (**D**) $FEF_{25–75}$ < LLN. Odds ratios (ORs) and 95% confidence intervals (CIs) represent the association between metal mixture indices and the likelihood of spirometry measures falling below the LLN. Models were adjusted for duration since the fire (in months), age, sex, smoking status, and BMI and restricted to spirometry tests meeting ATS/ERS quality grades A–C. Sample sizes differed by outcome: FVC < LLN (N = 737), FEV_1 < LLN (N = 790), and FEV_1/FVC < LLN and $FEF_{25–75}$ < LLN (N = 624). Time since the fire was inversely associated with FVC < LLN in this sensitivity analysis, consistent with possible improvement over time, although these cross-sectional results should not be interpreted as within-person recovery trajectories. Statistical significance is indicated as follows: +$p < 0.10$; *$p < 0.05$; **$p < 0.01$; ***$p < 0.001$.

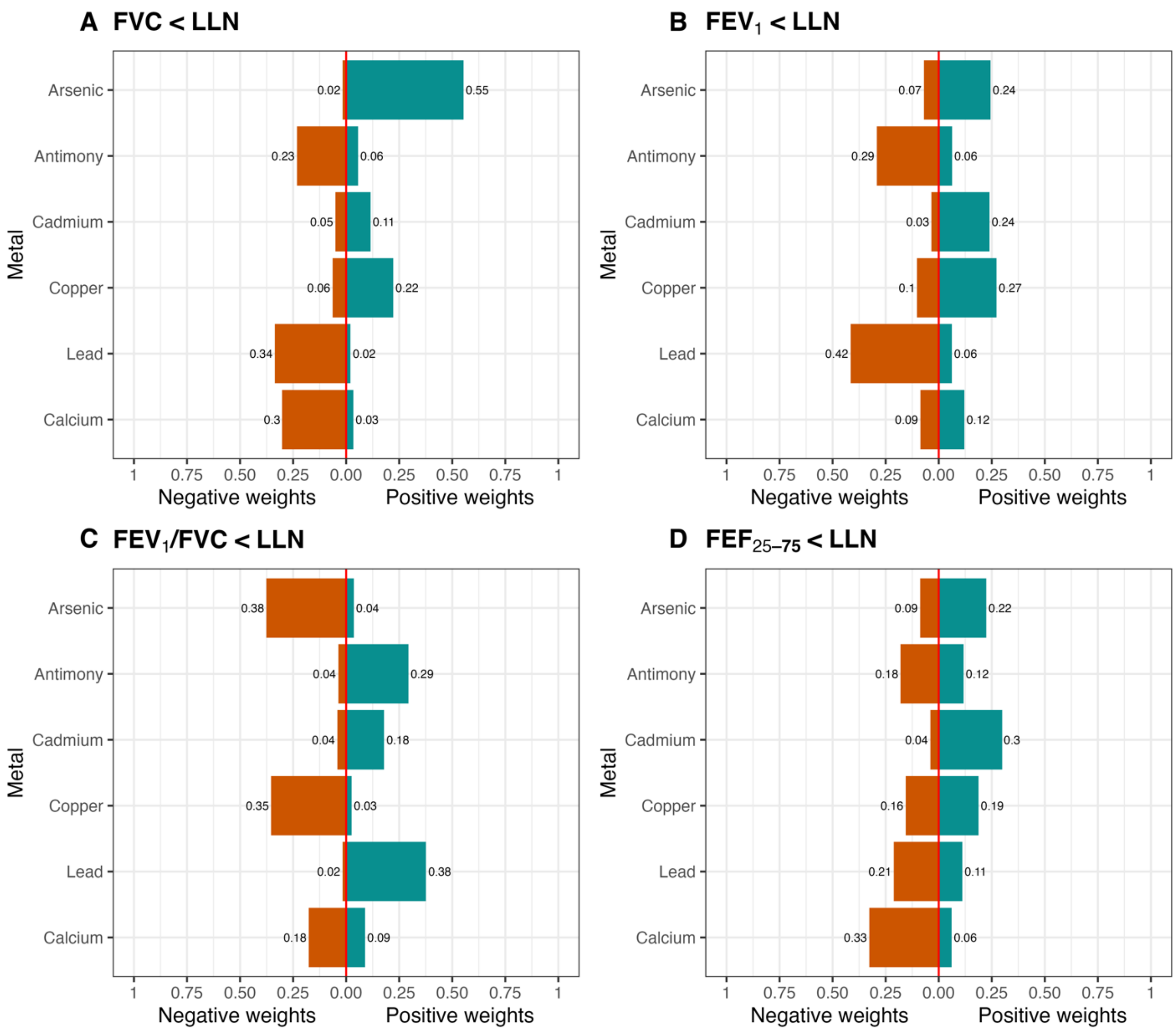

Figure S16. Sensitivity analysis of metal-specific weight contributions to pulmonary outcomes in dual-index weighted quantile sum (WQS) regression models controlling for time since the fire. Panels depict the relative contribution and directionality of individual metals associated with (**A**) FVC < LLN, (**B**) FEV_1 < LLN, (**C**) FEV_1/FVC < LLN, and (**D**) $FEF_{25–75}$ < LLN. Bars indicate estimated weights assigned to each metal within positive (right) and negative (left) mixture indices, with direction defined by the dominant absolute weight contribution. Models were adjusted for duration since the fire (in months), age, sex, smoking status, and BMI and restricted to spirometry tests meeting ATS/ERS quality grades A–C. Sample sizes differed by outcome: FVC < LLN (N = 737), FEV_1 < LLN (N = 790), and FEV_1/FVC < LLN and $FEF_{25–75}$ < LLN (N = 624).

References

1. K. Schilling, *et al.*, Pharmacokinetics of metal excretion following different doses of sodium EDTA infusion. *Metallomics* **17**, mfaf010 (2025).

2. R. W. Hornung, L. D. Reed, Estimation of Average Concentration in the Presence of Nondetectable Values. *Applied Occupational and Environmental Hygiene* **5**, 46–51 (1990).

3. B. Nermell, *et al.*, Urinary arsenic concentration adjustment factors and malnutrition. *Environmental Research* **106**, 212–218 (2008).

4. J. R. Kuiper, K. M. O'Brien, K. K. Ferguson, J. P. Buckley, Urinary specific gravity measures in the U.S. population: Implications for the adjustment of non-persistent chemical urinary biomarker data. *Environment International* **156**, 106656 (2021).

5. Centers for Disease Control and Prevention, NHANES Questionnaires, Datasets, and Related Documentation. (2012). Available at: https://wwwn.cdc.gov/nchs/nhanes/continuousnhanes/default.aspx?BeginYear=2011 [Accessed 13 November 2025].

6. I. T. Jolliffe, J. Cadima, Principal component analysis: a review and recent developments. *Philosophical Transactions of the Royal Society A: Mathematical, Physical and Engineering Sciences* **374**, 20150202 (2016).

7. S. Renzetti, C. Gennings, S. Calza, A weighted quantile sum regression with penalized weights and two indices. *Frontiers in Public Health* **11** (2023).

8. Q. An, *et al.*, Analysis of relationship between mixed heavy metal exposure and early renal damage based on a weighted quantile sum regression and Bayesian kernel machine regression model. *Journal of Trace Elements in Medicine and Biology* **84**, 127438 (2024).

9. J. F. Bobb, *et al.*, Bayesian kernel machine regression for estimating the health effects of multi-pollutant mixtures. *Biostatistics* **16**, 493–508 (2015).

10. B. L. Graham, I. Steenbruggen, Reply to Townsend: The American Thoracic Society/European Respiratory Society 2019 Spirometry Statement and Occupational Spirometry Testing in the United States. *American Journal of Respiratory and Critical Care Medicine* **201**, 1012–1012 (2020).

11. P. H. Quanjer, D. J. Brazzale, P. W. Boros, J. J. Pretto, Implications of adopting the Global Lungs Initiative 2012 all-age reference equations for spirometry. *European Respiratory Journal* **42**, 1046–1054 (2013).
